# Astrocytic uptake of neuronal corpses promotes cell-to-cell spreading of tau pathology

**DOI:** 10.1186/s40478-023-01589-8

**Published:** 2023-06-17

**Authors:** Tobias Mothes, Benjamin Portal, Evangelos Konstantinidis, Khalid Eltom, Sylwia Libard, Linn Streubel-Gallasch, Martin Ingelsson, Jinar Rostami, Maria Lindskog, Anna Erlandsson

**Affiliations:** 1 Department of Public Health and Caring Sciences; Molecular Geriatrics, Rudbeck Laboratory, Uppsala University, 752 37 Uppsala, Sweden; 2 Department of Medical Cell Biology, Uppsala University, Uppsala, Sweden; 3 Department of Immunology, Genetics and Pathology, Neuro-Oncology and Neurodegeneration, Uppsala University, Uppsala, Sweden; 4 University Health Network, Krembil Brain Institute, Toronto, Canada; 5 Department of Medicine and Tanz Centre for Research in Neurodegenerative Diseases, University of Toronto, Toronto, Canada

**Keywords:** Alzheimer’s disease, Tau, Astrocytes, Neurons, Cell-to-cell spreading, hiPSCs

## Abstract

Tau deposits in astrocytes are frequently found in Alzheimer’s disease (AD) and other tauopathies. Since astrocytes do not express tau, the inclusions have been suggested to be of neuronal origin. However, the mechanisms behind their appearance and their relevance for disease progression remain unknown. Here we demonstrate, using a battery of experimental techniques that human astrocytes serve as an intermediator, promoting cell-to-cell spreading of pathological tau. Human astrocytes engulf and process, but fail to fully degrade dead neurons with tau pathology, as well as synthetic tau fibrils and tau aggregates isolated from AD brain tissue. Instead, the pathogenic tau is spread to nearby cells via secretion and tunneling nanotube mediated transfer. By performing co-culture experiments we could show that tau-containing astrocytes induce tau pathology in healthy human neurons directly. Furthermore, our results from a FRET based seeding assay, demonstrated that the tau proteoforms secreted by astrocytes have an exceptional seeding capacity, compared to the original tau species engulfed by the cells. Taken together, our study establishes a central role for astrocytes in mediating tau pathology, which could be of relevance for identifying novel treatment targets for AD and other tauopathies.

**Supplementary Information:**

The online version contains supplementary material available at 10.1186/s40478-023-01589-8.

## Introduction

Alzheimer’s disease (AD) is the most common age-related neurodegenerative disorder, accounting for an estimated 60–80% of all dementia cases [1]. The key neuropathological hallmarks of AD are extracellular amyloid-β (Aβ) plaques, intracellular neurofibrillary tangles (NFTs) and chronic neuroinflammation. Although these characteristics have been known for decades, the cellular mechanisms behind AD propagation remain elusive. Since the proposition of the amyloid hypothesis by Hardy and Higgins in 1992, the majority of AD research has been dedicated to Aβ and its effects on neurons [2-4]. However, with a deeper understanding of disease complexity and heterogeneity, glial cells and tau pathology have recently received much attention. The microtubule-associated protein tau, was first described in 1975 and was discovered as the main component of NFTs a decade later [5-7]. Since then, many post-translational modifications (PTMs) have been reported for tau in the AD brain, with phosphorylation being the most commonly described [8]. In addition to NTFs, tau inclusions are frequently found in astrocytes in both AD and other tauopathies [9].

Astrocytes are the most numerous glial cell type, hosting a wide range of functions that are crucial for maintaining brain homeostasis [10]. In neurodegenerative diseases, their role is complex, as they seem to both alleviate and contribute to pathology. We have previously shown that astrocytes accumulate large amounts of aggregated Aβ and α-synuclein (α-syn), but not the monomeric form of the proteins [11, 12]. This intracellular storage of pathological proteins results in severe cellular stress, indicated by lysosomal and mitochondrial deficiencies [11-16]. Subsequently, the stressed astrocytes respond by contacting nearby cells via TNTs, enabling cell-to-cell transfer of protein aggregates and major histocompatibility complex II (MHCII) complexes [12, 17, 18]. Since astrocytes do not express tau, astrocytic tau inclusions are considered to be of neuronal origin [9, 19-21]. However, the mechanism behind the appearance of these tau deposits and their relevance for disease progression is unclear. Our results demonstrate that astrocytes, in addition to aggregated proteins, effectively ingest and accumulate dead cells, both in vitro and in vivo [22, 23]. Incomplete digestion of cell corpses by astrocytes might constitute a source of pathogenic aggregates. The aim of the present study was to delineate the role of astrocytes in cell-to-cell propagation and seeding of tau pathology, using a human iPSC-based disease model.

## Methods

### Culture of human iPSC-derived astrocytes

Human astrocytes were differentiated from neuroepithelial-like stem (NES) cells, produced from human induced pluripotent stem cells (iPSCs, Cntrl9 II cell line) [24]. To generate astrocytes, NES cells were cultured in Advanced DMEM/F12 (Thermo Fisher, 12634–010) supplemented with 1% penicillin–streptomycin (Thermo Fisher, 15140–122), 1% L-glutamine (Thermo Fisher, 25030–024), 1 × B27 (Thermo Fisher, 11530536) and 1 × non-essential amino acids (Thermo Fisher, 11140050). The following factors were added to the medium right before use: 8 ng/ml bFGF (Thermo Fisher, 13256029), 10 ng/ml heregulin beta-1 (Sigma, SRP3055), 10 ng/ml activing A (Peprotech, 120-14E), 200 ng/ml IGF-1 (Sigma, SRP3069). From week three of differentiation, 20 ng/ml of CNTF (Thermo Fisher, PHC7015) was also included. A full medium change was performed every other day for the duration of the differentiation. Cells were cultured in cell culture flasks (Sarstedt) coated with 100 µg/ml poly-L-ornithine (Sigma, P3655) and 50 µg/ml laminin (Sigma, L2020), and seeded for experiments at 5 000 cells/cm^2^. Trypsin–EDTA 4% (Thermo Scientific, 10779413) was used for passaging the cells and the cells were differentiated for 28 days, prior to the start of experiments.

### Culture of human iPSC-derived neurons

Human neurons were generated using the same NES cells as for the astrocytes [25]. The NES cells were plated at 40 000 cells/cm^2^ in cell culture flasks (Sarstedt) coated with 100 µg/ml poly-L-ornithine (Sigma, P3655) and 50 µg/ml laminin (Sigma, L2020) and cultured for five days in neuronal differentiation medium: DMEM/F12 + Glutamax (Fisher Scientific, 31331028) supplemented with 1% N2 (Fisher Scientific, 11520536), 1% penicillin–streptomycin (Thermo Fisher, 11548876), 1 × B27 (Thermo Fisher, 17504044). During this period, the medium was fully replaced every other day. The cells were detached from the flask using 1 × TrypLE (Thermo Fisher, 12563029), re-plated for experiments at a density of 20 000 cells/cm^2^ (coating was performed like before but with 5 × laminin concentration) and cultured for another five days (until d10). During this period, half of the medium was replaced every other day. From day ten and onwards the neuronal differentiation medium was mixed 1:1 with complete neurobasal medium, consisting of Neurobasal medium (Thermo Fisher, 21103049) supplemented with 1% penicillin–streptomycin (Thermo Fisher, 11548876), 1 × B27 and 1 × GlutaMAX (Thermo Fisher, 35050038). Neurons directly exposed to Tau-F were cultured for 28 days in total (14 days in the presence of Tau-F). The neurons in co-culture with astrocytes were kept in culture for 66 days (28 days in co-culture with astrocytes).

### Co-culture of human neurons and astrocytes

For co-culture experiments, astrocytes (differentiation day 28) were added to the neuronal cultures (differentiation day 38) at a 10:1 neuron:astrocyte ratio. Once in co-culture, the cells were cultured for additional four weeks in the 1:1 medium described above. Throughout this period, half of the medium was replaced twice a week.

### *Production of *in vitro* tau aggregates*

Human tau fibrils (Tau-F) were generated using recombinant human 441-tau monomers (Anaspec, AS-55556). Monomers were initially dissolved (3 mg/ml) in 100 mM MES hydrate (Sigma, M2933) buffer, pH 6.5, containing 10 µM of 1,4-Dithiotheitol (Sigma, D0632) and 16.25 µM heparin (Sigma H3149) and incubated on slow shake at 37 °C for seven days. Tau aggregates were then centrifuged at 20879xg for 30 min, 4 °C and the pellets re-suspended in Phosphate-buffered saline, PBS (1 mg/ml). When required, the fibrils were labeled using Amersham Cy3-labeling kit (Amersham, PA33000) according to the manufacturer’s instructions. Fibrils were used immediately or stored at − 70 °C. Prior to experiment, the fibrils were sonicated at 20% amplitude, 1 s on/off for 30 s, using a Sonics Vibra Cell sonicator. The tau aggregation protocol was adapted from Goedert et al. [26].

To produce tau oligomers (Tau-O), we used the same tau monomers and buffers as for the fibrils, but the tau and heparin concentrations were reduced to 1 mg/ml and 5 µM, respectively. Incubation took place on a shaker for five hours. Oligomers were immediately used or stored at − 70 °C.

### Tau extraction from human AD temporal cortex

The procedure was performed as previously described [27]. Human AD temporal cortex was homogenized in 10 mM Tris–HCL, pH 7.5, 0.8 M NaCl, 10% sucrose and 1 mM EGTA (10 × v/w) using precellys evolution, 6800 RPM 3 × 20 s cycles with 30 s pauses. Brain homogeneates were brought up to 2% N-Lauroylsarcosine and incubated for 30 min at 37 °C on mild shake. Homogenates were centrifuged at 7000 g for 10 min at 4 °C and the pellet was discarded. Following the supernatant was centrifuged at 100,000*g* for 60 min (4 °C) using a Hitachi CS150NX ultra-centrifuge with a S50ST rotor. The pellet was resuspended in 1 × PBS to constitute 1 g starting tissue/ml of brain derived tau (BDTau-F).

### Transmission electron microscopy of synthetic and brain derived tau aggregates.

Characterization of Tau-F, Tau-O and BDTau-F was performed by transmission electron microscopy (TEM) using negative staining. Tau-F were diluted 1:10 in distilled H2O and placed on a formvar and carbon coated 200-mesh copper grid (Ted Pella). The sample was directly stained with 2% uranyl acetate. Dried grids were examined by TEM (FEI Tecnaii G2) operated at 80 kV with an ORIUS SC200 CCD camera and Gatan Digital Micrograph software (both from Gatan Inc.).

### Tau exposure of cell cultures

Astrocytes were actively exposed to sonicated Tau-F (Cy3-labeled or unlabeled) for 24 h, 3 days or 7 days before being fixed or lysed. Other cultures were kept in tau-free medium for additional time after being thourghly washed at 24 h or 3d (24 h + 6d, 3d + 4d, 3d + 8d, 3 + 11d, 3d + 12d and 3 + 25 d). Tau-F media concentrations were 200 nM or 480 nM. For co-culture experiments, the astrocytes were pre-exposed to 200 nM Tau-F for three days before being washed and transferred to the neuronal cultures.

Tau exposure of neurons was performed in the same way as described for the astrocytes above, with medium concentrations of 50 nM, 200 nM Tau-F or 50 nM Tau-O. From the exposure time point onwards, the half media change was only performed twice a week.

### Exposure of astrocytes to apoptotic neurons

Tau exposed neurons and untreated, control neurons were washed three times with PBS and then exposed to one 480 mJ UV-burst in a GS Gene linker UV chamber (BioRad). Directly after the burst, astrocytes were added to the apoptotic neurons at a ratio of 1:3 (astrocytes: apoptotic neurons). The cells were cultured for three and seven days to allow astrocytic uptake of cell corpses and debris.

### Time-lapse microscopy

Time-lapse experiments were performed at 37 °C in humidified 5% CO2 in air, using a Nikon Biostation IM Live Cell Recorder (Nikon). Cells were cultured in time-lapse culture dishes (VWR, 391–0256) and the cells were pre-exposed to 200 nM Cy3Tau-F for 3 days and washed prior to starting the recording. Images were captured every 10 min, with the exception of images presented in Fig. 5b, which were captured every 3 min.

### BioTracker transmission assay

To evaluate transfer efficiency of tau aggregates, one population of astrocytes was exposed to Cy3Tau-F for 3 days (donor) before being washed and put in co-culture with a second population of astrocytes (acceptor) labeled with BioTracker (BioT) 490 Green Cytoplasmic Membrane Dye (Sigma, SCT106). The BioTracker dye was used according to the manufacturer’s recommendations. The Cy3 signal of acceptor astrocytes was then measured as described in the image analysis section.

### Tau seeding assay

The tau RD P301S Biosensor HEK cell line (ATCC, CRL-3275) was used to evaluate seeding capacity of Tau-F and BDTau-F. The HEK cells were cultured according to the ATCC’s recommendations. Astrocytes were exposed to 200 nM Tau-F or BDTau-F for 3 days before being washed and subjected to four additional days of incubation in Tau-free medium (3d + 4d). Conditioned medium from astrocytes (ACM) was fortified with 1% lipofectamine 3000 (Fisher Scientific, L3000015) and 10% fetal bovine serum, (FBS) and added to the biosensor cells for 48 h. the FRET signal (YFP emission due to 405 nm laser excitation) was captured using a LSM700 confocal microscope. Negative control (medium from control astrocytes) and positive control (Tau-F in medium) were both fortified with lipofectamine and FBS as described above.

### Western blot (WB)

For lysis, cells were incubated in ice cold lysis buffer (20 mM Tris pH 7.5, 0.5% Triton X-100, 0.5% Deoxycholic acid, 150 mM NaCl, 10 mM EDTA, 30 mM Na_4_O_7_P_2_, supplemented with 1 × Halt Protease Inhibitor Cocktail (Thermo Scientific, 78,430)) for 10 min before being scraped off the plate with a cell scraper (Thermo Fisher, 99,002). The cell samples were transferred to LO-bind tubes and incubated on ice for 30 min before being centrifuged at 28 000 g for 30 min (4 °C). The lysates were separated from pellets and stored in − 70 °C until use. Total protein concentrations of cell lysates were determined using Pierce BCA protein assay kit (Thermo Scientific, 23225) according to the manufacturer’s instructions. Samples (18 µg) were denatured by incubating with BoltTM sample reducing agent (Invitrogen, B00009) in LDS sample buffer (Invitrogen, NP0007) for 5 min at 95 °C. The samples were then loaded on a 4–12% Bis–Tris Plus Gel (Invitrogen, NW04125BOX) with 5 µl Chameleon Duo (LI-COR, 928–60000) protein ladder and run for 20 min at 200 V in MES SDS running buffer (Thermo Fisher, B0002). Transfer to PVDF membrane was performed in BoltTM transfer buffer (Invitrogen, BT00061), supplemented with 10% methanol and 0.1% BoltTM antioxidant (Invitrogen, BT000) for 1 h at 20 V. The total protein in each lane was measured by the no-stain Protein Labeling Reagent (Thermo Fisher, A44449) followed by BIO-RAD ChemiDoc XRS + reading. Blocking was performed with 5% bovine serum albumin (BSA) in Tris Buffered Saline-Tween20 (TBS-T) for 1 h at room temperature (RT). The membrane was then incubated with primary antibodies (listed in Additional file 8: Table.S1) diluted in 5% BSA TBS-T at 4 °C overnight and secondary antibodies (goat anti-rabbit and anti-mouse DyLight 680, and goat anti-rabbit and anti-mouse DyLight 800, diluted 1:20,000 in 5% BSA, TBS-T) for 1 h at RT. The signal was analyzed using an SA Odyssey (LI-COR). Band intensity was measured using the ImageStudio (LI-COR) or ImageLab (BIO-RAD) software. Each band was normalized against the lane intensity of the total protein labeling signal. Each experiment was repeated three times.

### Sandwich ELISA

Corning high-binding 96-well plates (VWR, 734–1624) were coated with 1 µg/mL of the tau antibodies BT2 or T46 for 24 h at 4 °C. Then, the plates were blocked for 2 h with 1% BSA at RT. To denature protein aggregates and/or vesicles, the conditioned media samples were fortified with 1% SDS and heated to 95 °C for 5 min prior to loading onto the plate. Samples were incubated for 24 h at 4 °C. For detection the tau antibody Tau-12 was first biotinylated using EZ-Link biotinylation kit (Thermo, A39257) according to the manufacturers instructions. 1 µg/mL of the biotinylated Tau-12 antibody was added and the plate was incubated for 2 h at RT on mild shake. Then, aqueous TMB substrate (Lumiradx, 331177) was added for ~ 10 min before addition of 1 M H_2_SO_4_ to halt the reaction. Detection was performed using the Infinite M200 pro ELISA reader. Each experiment was repeated three times.

### Immunocytochemistry (ICC)

Cells were fixed using 4% paraformaldehyde (Sigma) in PBS for 15 min at RT and washed twice with PBS. Blocking and permeabilization were performed by incubation in 5% normal goat serum (NGS) and 0.1% Triton X-100 or Saponin in PBS for 30 min at RT. Primary antibodies (listed in Additional file 8: Table.S1, diluted in 0.5% NGS 0.1% Triton X-100/saponin in PBS) were added overnight at 4 °C. The cells were washed 3 × 10 min with PBS before incubation with secondary antibodies diluted in 0.5% NGS and 0.1% Triton X-100 or saponin in PBS for 1 h at 37 °C. AlexaFluor goat-anti mouse, rabbit or chicken; 488, 555 or 647 (1:200, Molecular Probes) were used as secondary antibodies. Cells were washed 3 × 5 min with 1 × PBS and mounted on microscope slides using Ever Brite Hardset Mounting medium with or without DAPI (VWR, 23004 and 23003). Images were captured using a fluorescence Observer Z1 Zeiss microscope and a confocal LSM700 microscope.

### Image analysis

Fluorescent images used for quantification were captured with the Observer Z1 Zeiss microscope (40 × objective). For the biosensor FRET quantification (YFP emission due to 405 nm laser excitation) images were captured using a LSM700 confocal microscope. The z-stack images (25–30 images/z-stack) were compiled as a composite for max intensity. Each experiment were repeated three times and 12–15 randomly captured images were analysed per experiment. All quantifications were performed in ImageJ.

For the Cy3Tau-F inclusions we developed a specific ImageJ macro. First, the region of interest (ROI) was determined (based on a cellular marker) to only include intracellular Cy3 in the measurement. Next, the corresponding Cy3 images were analysed based on the following steps: set scale, convert to 16-bit, subtract background, set threshold (the same threshold was used for all time-points), clear outside (everything outside of the ROI), set measurements and analyze particles. In each image, Cy3tau deposits were assessed by measuring the total area, the number of particles and the sum of the integrated densities (area x mean intensity of each Cy3tau deposit), normalized to the number of living cells (identified by DAPI staining). Since various sized Cy3 tau inclusions were observed, the ImageJ macro was adjustable to quantify different types of inclusions separately. Judging by the area measurements of the various particles, the cut-off limit for the Cy3Tau inclusions were set to 5–200 pixels (small), 201–2000 pixels (medium) and 2001-infinity pixels (large).

Quantification of the biosensor YFP IntDen was performed in the same way as described for Cy3 signal. YFP IntDen was normalized to the total cell area/image (instead of DAPI since it would interfere with the CFP/YFP emission). Cell area was manually drawn for each image and measured. To allow blinding, this process was performed on corresponding phase contrast images.

Cell branching was measured using a custom macro. The analysis was performed on GFAP-images using the following steps: convert to 8-bit, set threshold, find edge, Gaussian blur, convert to mask, skeletonize, create selection. The new selection was then put through several interactions of dilation and erosion to get the best fit possible. Branch points were calculated when pixels were in contact with 3 or more other pixels.

### Patch clamp experiments

Cells grown on glass coverslips were placed in a patch clamp chamber (#RC-27, Warner Instruments, CT, USA) and perfused with an extracellular solution containing: 110 mM NaCl, 1 mM NaH_2_PO_4_, 4 mM KCL, 25 mM 4-(2-hydroxyethyl) piperazine-1-ethanesulfonic acid (HEPES), 100 mM glucose, 1.2 mM MgCl_2_ and 1.5 mM CaCl_2_ (pH 7.4) at a rate of 2–3 ml per min and a temperature of 32 °C. Borosilicate glass pipettes with a tip resistance of 3–7 MOhm were used for patching the cells. The glass pipettes were filled with a solution containing: 110 mM K-gluconate, 10 mM KCl, 4 mM Mg-ATP, 10 mM Na_2_-phosphocreatine, 0.3 mM Na-GTP, 10 mM HEPES and 0.2 mM ethylene glycol tetra acetic acid (EGTA) (pH 7.2–7.4; 270–290 mOsm). Resting membrane potential was measured immediately after opening the patched cell. Excitatory Post Synaptic Currents (sEPSCs) were recorded, as well as action potential independent synaptic currents (mEPSCs) occuring from spontanous fusion of presynaptic vesicles recorded in the presence of 0.5 μM of tetrodotoxin (TTX) to block action potentials. Access resistance was monitored throughout the recordings, only data displaying < 30% variation were included. Data acquisition was performed using an Ag/AgCl electrode, a Multiclamp 700B amplifier digitized with Digidata 1440A (Molecular Devices CA, USA). Recordings were monitored using the Clampex software 10.0 (Molecular Devices).

### Analysis of excitatory post synaptic potentials (EPSCs)

Traces were analyzed with the Easy Electrophysiology software (v2.3.3-beta). Event detection was based on a template fitting method. We first used probe recordings (three recordings of one minute each) from primary neuronal cultures to generate and refine a template from representative events corresponding to an average of ten to twenty detected excitatory EPSC. Event detection in iPSC derived neuron cultures was then run on a semi-automatic method: each event detected fits the previously generated template. When the amplitude of a detected event was the same as the template one, the event was saved for further analysis. A minimum amplitude threshold of 10 pA was applied. All events that were detected between 0 and 10 pA were discarded. Each recording was analyzed in a period of 1 min.

### Statistical analyses

All statistical analyses, with an exception for the electrophysiology data, was performed in Graphpad Prism (v.9.3.1). The data sets were initially analyzed using the D’Agostino-Pearson omnibus and the Shapiro–Wilk normality tests prior further analysis. Datasets that passed both normality tests were analyzed using one-way ANOVA (for datasets with multiple groups) or unpaired t-test when comparing only two groups. Data sets where any group failed the normality tests were instead analyzed using the Kruskal–Wallis non-parametric test for more than two groups and the Mann–Whitney u-test for comparisons with only two groups. P-values are presented as following; *p < 0.05, **p < 0.01, ***p < 0.005, ****p < 0.0001.

Regarding electrophysiology data, statistical analysis was conducted in IBM® SPSS® Statistics (v28). For every measured variable, a Kolmogorov–Smirnov test of normality and a Levene test for homogeneity of variances were conducted to determine the type of statistical test. Single rank signed Wilcoxon test and a Kruskal–Wallis test were used to analyze the resting membrane potential. Kruskal–Wallis tests were used to analyze the frequency, amplitude, rise time and decay time of both sEPSCs and mEPSCs. A Bonferroni multiple comparison test was applied when the result of the Kruskal–Wallis test allowed it. We noticed a very high variation within groups, especially for frequency of both sEPSCs and mEPSCs, thus the R package “cvequality” (v0.1.4) was used to analyze potential differences in the coefficient of variation [28]. Asymptotic test for equality of coefficient of variation revealed no differences across groups for the frequency of sEPSCs (χ^2^ = 0.620, *p* = 0,959) [29]. However, coefficient of variation in amplitude of sEPSCs were statistically different across groups (χ^2^ = 12.44, *p* = 0.014). The same analysis revealed no differences in the coefficient of variation in the frequency of mEPSCs nor amplitude of mEPSCs (χ^2^ = 2.15, *p* = 0.706; χ^2^ = 5.74, *p* = 0.218 respectively for frequency and amplitude of mEPSCs). Overall, this reveals that the variation is similar between the groups, regardless the considered variable. Whilst usually big variations mask potential statistical effect, it seems that in our study such variation did not, pointing out the robust aspect of our statistical analysis. For every test, p-values below 0.05 were considered significant. More detail is outline in Additional file 9: Table.S2.

## Results

### Cellular model to study the role of astrocytes in tau pathology

**Fig. 1:**
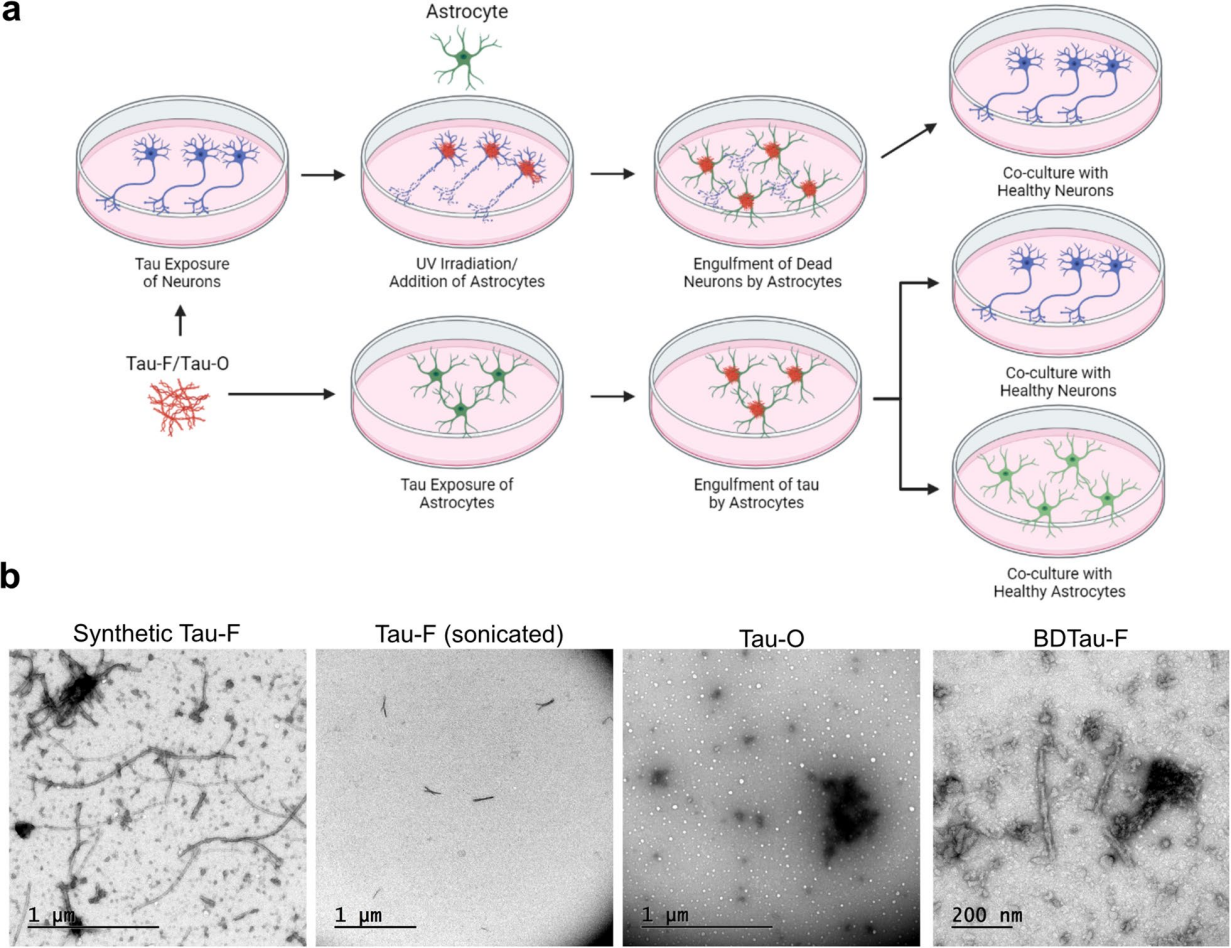
A human iPSC-based disease model to study cell-to-cell propagation of tau pathology. **a** Schematic outline of the experimental set-up. Neurons were exposed to tau aggregates and cultured in tau-free medium for additional time, followed by a UV-burst. Astrocytes were co-cultured with the apoptotic neurons to allow ingestion of the cell corpses and neuronal debris. Finally, the phagocytic astrocytes were introduced to a second cell culture consisting of healthy neurons. In parallel experiments, astrocytes were directly exposed to tau aggregates and then co-cultured with either healthy astrocytes or healthy neurons. **b** TEM images of synthetic tau fibrils, their sonicated counterpart (Tau-F), synthetic tau oligomers (Tau-O) and human AD brain derived tau (BDTau-F)

In order to investigate if astrocytes promote cell-to-cell spreading of toxic tau aggregates following ingestion of dead neurons we designed an experimental set-up based on hiPSC-derived cell cultures (Fig. 1 and Additional file 2: Fig.S1). First, we induced robust tau pathology in human neurons. For this purpose, we exposed hiPSC-derived neurons to tau seeds in form of in vitro produced tau oligomers (Tau-O) or sonicated tau fibrils (Tau-F) (Fig. 1b).

### Exposure to tau fibrils induces tau pathology in human neurons

**Fig. 2:**
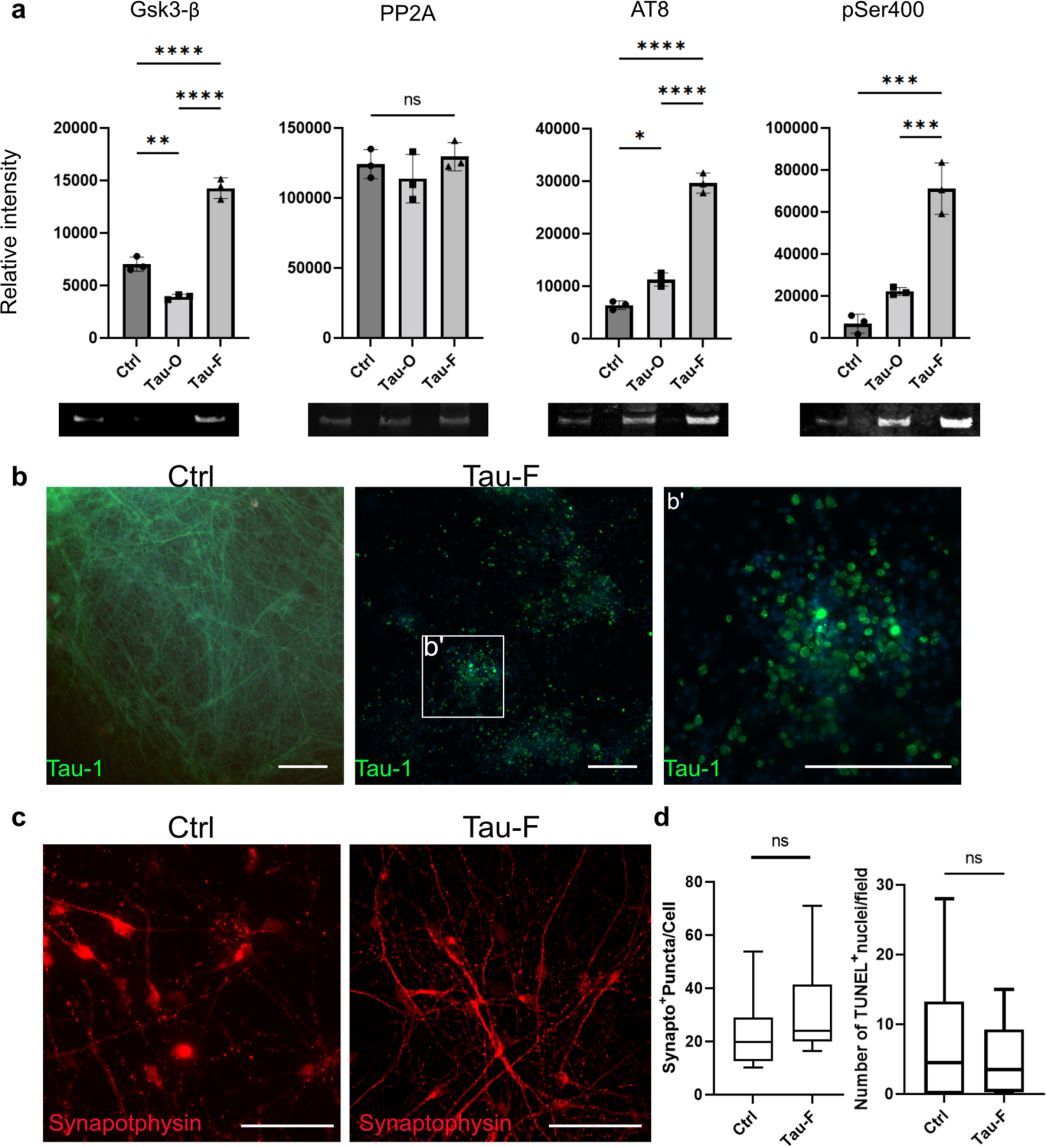
Sonicated tau fibrils induce distinct tau pathology in human iPSC-derived neurons. **a** WB analysis of cell lysates from control, Tau-O and Tau-F exposed neurons for Gsk3-β, PP2A, AT8 and pSer400 (relative to total protein). **b** Representative fluorescence images of total tau (Tau-1) in control neurons and Tau-F exposed neurons. Tau-F exposed neurons display a distinct tau-pattern with spherical deposits. Scale bars = 200 µm. **c** Control and Tau-F exposed neurons stained for synaptophysin (co-staining with WGA is presented in Additional file 4: Fig. S3a). Scale bars = 50 µm. **d** Quantification of synaptophysin (Syn) positive puncta and TUNEL positive nuclei (representative images are shown in Additional file 4: Fig. S3b) normalized to the total number of cells. Error bars represent min/max values. One-way ANOVA and t-test were used for graphs in (**a**) and (**d**) respectively. *p < 0.05, **p < 0.01, ***p < 0.005, ****p < 0.0001

To determine if the synthetic tau aggregates induced tau pathology in hiPSC-derived neurons, the cells were cultured for two weeks following tau exposure. WB analysis of cell lysates was performed with antibodies against pathological tau species, as well as glycogen synthase-kinase 3 beta (Gsk3-β) and protein phosphatase 2 A (PP2A), both of which are enzymes involved in tau phosphorylation (Fig. 2a). For instance, GSK3-β phosphorylates serine 202 and threonine 205 (central region of tau), and these phosphorylation forms are recognized by the widely used AT8 antibody. Protein phosphatase 2A (PP2A) is considered to oppose the effect of GSK3-β by dephosphorylating many of its targets. Exposure to 50 nM Tau-F from differentiation day 14 to day 28 (Fig. 2a) lead to a distinct increase in the expression of GSK3-β, compared to untreated control neurons. Parallel neuronal cultures that were exposed to 50 nM Tau-O, showed the opposite result, with decreased levels of GSK3-β compared to control cells. No statistically significant difference between the groups was observed for PP2A (Fig. 2a). Notably, WB analysis using the p-tau-antibodies AT8 and pSer400 demonstrated that Tau-F exposure resulted in a more pronounced tau pathology, compared to Tau-O (Fig. 2a). The reason for that is probably the high toxicicity of tau-O, i.e. the neurons die before the seeding have time to induce tau phosphorylation. Based on these data, we decided to focus on Tau-F exposure for inducing tau pathology. The effect of tau exposure was further investigated with immunocytochemistry of the neuronal monocultures. The pattern in native tau protein, detected with the total tau antibody tau-1, also changed (Fig. 2b), indicating a redistribution of native tau protein in neurons exposed to Tau-F. To exclude that this effect was due to increased cell death or overall neuronal dysfunction, we also performed TUNEL analysis and synaptophysin staining, but were unable to detect any differences in viability or synapse patterns (Fig. 2c, d). In contrast to glial cells, neurons are not highly phagocytic cells. Hence, we sought to verify whether any of the added protein was taken up by the neurons. Visualisation using 3D z-stacks confirmed that although the majority of the Cy3Tau-F added to the neurons remained extracellular, a proportion of the tau aggregates was clearly internalized (Additional file 3: Fig. S2).

### Astrocytes store dead cells and protein aggregates

**Fig. 3:**
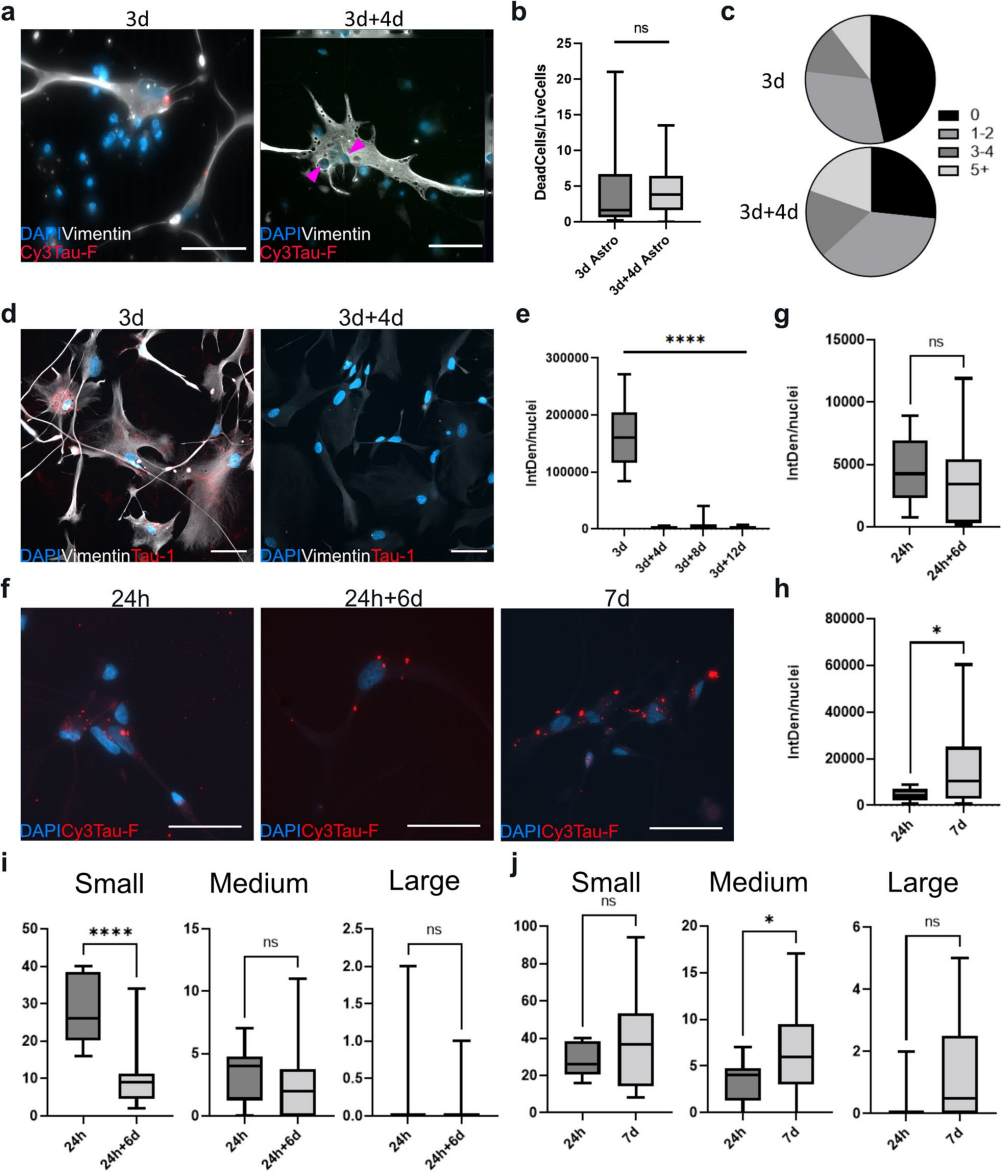
Astrocytes ingest dead cells and tau aggregates that are stored rather than degraded. **a** Astrocytes introduced to a culture of apoptotic neurons engulf and accumulate whole dead cells. Pink arrows indicate the condensed nuclei of dead neurons located inside an astrocyte. **b** Quantification of the total number of condensed nuclei, normalized to the total number of living astrocytes/field of view. **c** The proportion of astrocytes containing 0, 1–2, 3–4 or 5 + condensed nuclei at each time point/field of view. **d** Representative images (3d and 3d + 4d) of astrocytes exposed to unlabeled Tau-F, stained with the total tau antibody Tau-1. **e** Quantification of the Tau-1 signal, presented as IntDen/nuclei. **f** Representative images of astrocytes exposed to Cy3Tau-F for 24 h, 24 h + 6d and 7d. **g** Quantification of the Cy3-signal at 24 h and 24 h + 6d (degradation). **h** Quantification of the Cy3-signal at 24 h and 7d (accumulation) **i** The total number of small, medium and large tau deposits/live astrocytes at 24 h respective 24 h + 6d (j) and at 24 h respective 7d. Small deposits (0.164µm^2^- 16.4µm^2^), medium deposits (16.5µm^2^- 328µm^2^) large deposits (328 µm^2^-infinity). Scale bars = 50 µm. Error bars represent min/max values. T-test was used for the graphs in b, g and h and for the graph in e, Kruskal–wallis test was used. For all graphs in i-j, Mann–Whitney U test was used. *p < 0.05, **p < 0.01, ***p < 0.005, ****p < 0.0001

We have previously shown that human astrocytes effectively ingest large amounts of aggregated α-syn and Aβ. However, compared to microglia, astrocytes are exceptionally bad at degrading the engulfed material [18]. To clarify if this extends to neuronal debris, apoptosis of neurons was induced by an UV-burst. Immediately after the burst, hiPSC-derived astrocytes were introduced to the apoptotic neuronal cultures. To estimate the degrading potential of astrocytes, the total number of condensed nuclei (dead neurons) in relation to living astrocytes were quantified at 3 and 7 days (Fig. 3a). There was no significant change in the amount of condensed nuclei between the two time points (Fig. 3b), indicating that the astrocytes did not degrade the neuronal cell corpses to any notable degree within the time frame. At day 3, approximately half of the astrocytes had engulfed at least one dead cell but less than 25% contained more than two cells. At day 7, the proportion of astrocytes with no cell corpses had significantly decreased, indicating that they continuously ingest dead cells that remain on the culture dish, but do not effectively degrade the cell corpses (Fig. 3c).

We next investigated if astrocytes accumulate synthetic Tau-F in a similar way as they do with cell corpses. With the intention to detect tau deposits with the total tau antibodies we exposed astrocytes to unlabeled Tau-F. However, we noticed that the IntDen signal (signal intensity*area of signal) decreased directly after the wash at 3 days and quantification verified that there was almost no signal at 3 + 4d, 3 + 8d or 3 + 12d (Fig. 3d, e). Our previous studies of α-syn and Aβ in human astrocytes have shown that astrocytes modify ingested protein aggregates, and thereby make them difficult to detect with antibodies, although the aggregates are not degraded. To confirm if astrocytes handle aggregated tau in a similar way, Cy3-labeled Tau-F were added to the astrocytes. Analysis of the Cy3 signal indicated extensive Tau-F uptake already after 24 h (Fig. 3f). After 7 days of continuous Tau-F exposure, the signal had increased, demonstrating that astrocytes constantly ingest Tau-F when it is present (Fig. 3f, h). Astrocytes that were washed 24 h after the Tau-F addition and then cultured in Tau-F free medium for additional 6 days (24 h + 6d) still displayed Cy3 signals, illustrating that the cells indeed store a high proportion of the engulfed Tau-F (Fig. 3f, g). In addition to IntDen quantifications, we analyzed the size and over all amount of deposits present inside astrocytes at the different time points in order to assess how the astrocytes store the aggregates. In the cultures continuously exposed to Tau-F, the overall number of deposits remained similar, although there was a statistically significant increase in medium-sized aggregates (Fig. 3j). Comparison of 24 h and 24 h + 6d cultures revealed a significant reduction of small aggregates over time, but no significant changes in the number of medium sized or large aggregates (Fig. 3i). These results could be explained by the fact that the aggregates are brought closer together over the 6 d period. Hence, following, ingestion the tau aggregates are relocated inside the cell and finally end up in the “storage dumps” around the cell nuclei. Taken together, these data demonstrate that astrocytes take up extensive amounts of dead neurons and tau aggregates, which are stored and packed together rather than degraded. To verify this intracellular storage of tau, we performed WB on both lysates and pellet fraction from astrocytes exposed to tau-F for 14 days. The result confirm that a significant amount of tau is still present inside the cells at this time point (Additional file 5: Fig.S4).

### Tau-F induces an inflammatory response in astrocytes

**Fig. 4:**
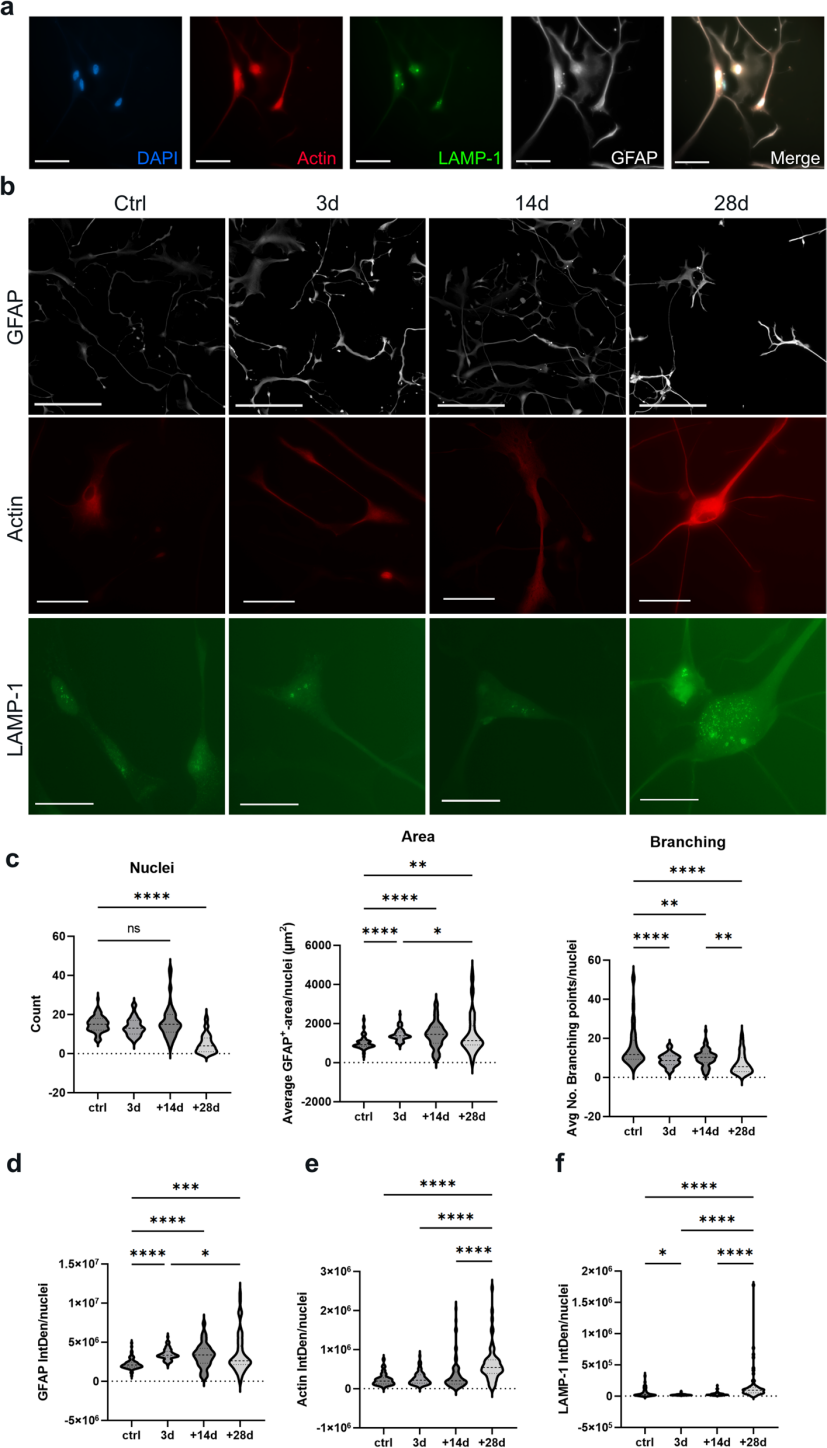
Long-term Tau-F exposure of astrocytes affect their viability and reactivity. **a** Astrocytes exposed to Tau-F, co-labelled for LAMP-1, GFAP, Actin and DAPI. **b** Representative images of astrocytes exposed to Tau-F for 3 days, 14 days and 28 days, stained for GFAP, Actin and LAMP-1. **c** Quantification of total astrocyte number, average cell area and number of branch points per cell. **d** Quantification of IntDen/cell for GFAP. **e** Quantification of IntDen/cell for actin. **f** Quantification of IntDen/cell for LAMP-1. The control group is combined from all time point since there was no statistically significant variation over time. For GFAP images, scale bars in a, are set to 50 µm and in b, 100 µm, 50 µm. For actin and LAMP-1, the scale bars = 25 µm. Statistical analysis in c-f was performed using Kruskal–Wallis test. *p < 0.05, **p < 0.01, ***p < 0.005, ****p < 0.0001

To understand how astrocytes are effected by the tau deposits over time, we decided to extend the incubation time by another two weeks. Astrocytes exposed to 200 nM of Tau-F for 3 days and then fixed at 14 days (3d + 11d) or 28 days (3d + 25d) were stained for DAPI, glial fibrillary acidic protein (GFAP), actin and lysosomal-associated membrane protein 1 (LAMP-1) to evaluate cell number, reactivity, morphology and lysosomal changes, respectively (Fig. 4a, b). Quantification of the living nuclei revealed a 67% reduction in total cell number at day 28, compared to control cultures, indicating a latent toxic effect in astrocytes with tau deposits (Fig. 4c). Using live cell imaging, we demonstrated that by that point, dead astrocytes were engulfed by the remaining astrocytes in the culture, which subsequently increased their stress load further (Additional file 6: Fig.S5 a). Moreover, we noted a changed morphology of the Tau-F exposed astrocytes over time, with increased area, but reduced number of cellular processes (Fig. 4c). Quantification of GFAP and actin (Fig. 4d, e) immunostainings revealed an increased expression of both markers over time in Tau-F exposed cultures, compared to control cultures. LAMP-1 expression was initially decreased in Tau-F exposed astrocytes, but was higher by day 28, when the toxic effect of the tau accumulation became apparent (Fig. 4f), likely due to an overall increase of astrocytic debris at that time point.

### Astrocyte spread intracellular tau deposits to nearby cells

**Fig. 5:**
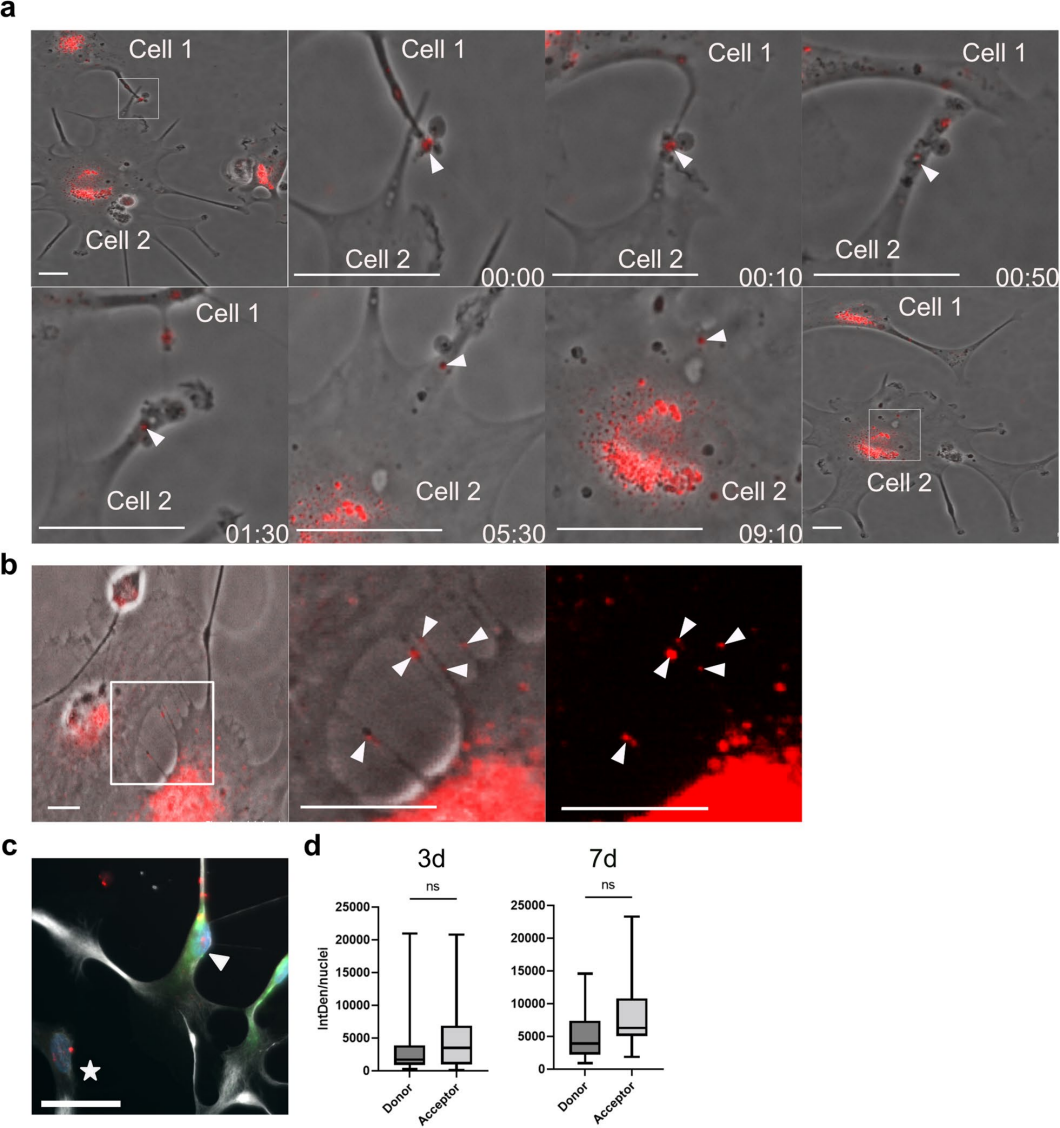
Astrocytes transfer ingested material between each other in an efficient manner. **a** Time-lapse microscopy of Cy3Tau-F exposed astrocytes, demonstrating a transfer event of Cy3Tau-F aggregates (white arrow) from cell 1 to cell 2 via direct membrane contact. Scale bars = 20 µm. **b** Time-lapse microscopy of Cy3Tau-F exposed astrocytes illustrating TNTs with tau inclusions (white arrows) between two astrocytes. Scale bars = 20 µm. **c** Co-cultures of astrocytes exposed to Cy3Tau-F (donor cells, white star) and unexposed (acceptor cells, white arrow) labeled with Biotracker-488 (green) demonstrated rapid distribution of tau deposits between the astrocytes. Scale bar = 50 µm. **d** Quantification of the IntDen Cy3 signal/cell in donor and acceptor astrocytes revealed an equal amount of intracellular tau aggregates in the two populations of astrocytes at 3 days and 7 days. T-test was used for statistical analysis in d. Scale bar = 20 µm

Knowing that astrocytes are capable of engulfing, storing and to some degree modifying Tau-F and cell corpses, we sought to investigate whether they also transmit tau deposits via direct contact and/or secretion. To investigate direct cell-to-cell transfer, we conducted time-lapse microscopy on Cy3Tau-F exposed astrocytes. Indeed, we frequently observed aggregates being transferred from one astrocyte to another, via membrane-to-membrane contact. Most of the time, Cy3Tau-F aggregates were located in proximal parts of an astrocyte, before they were transmitted to and subsequently transported towards the soma of a recipient cell (Fig. 5a). We also frequently observed formation of multiple TNTs, connecting two neigbouring astrocytes (Fig. 5b). Cy3Tau-F inclusions were observed traveling through the TNTs between cells (Additional file 6: Fig.S5b and Additional file 1: Movie 1). To evaluate how effectively astrocytes transmit the tau aggregates we performed co-cultures of unlabeled astrocytes exposed to Cy3Tau-F (donor cells) and untreated astrocytes (acceptor cells), labeled with the membrane dye (BioT) biotracker488. This futher verified that tau-containing astrocytes transfer tau inclusions to neighboring healthy cells (Fig. 5c). Quantifications of the IntDen signal in donor versus acceptor astrocytes after 3 and 7 days in co-culture demonstrated that the cells distributed tau deposits between each other very rapidly. After only 3 days, there was an equivalent amount of intracellular tau aggregates in the two astrocyte populations, which remained unchanged throughout the experiment (Fig. 5d).

### Tau accumulating astrocytes become toxic to neighboring neurons

**Fig. 6:**
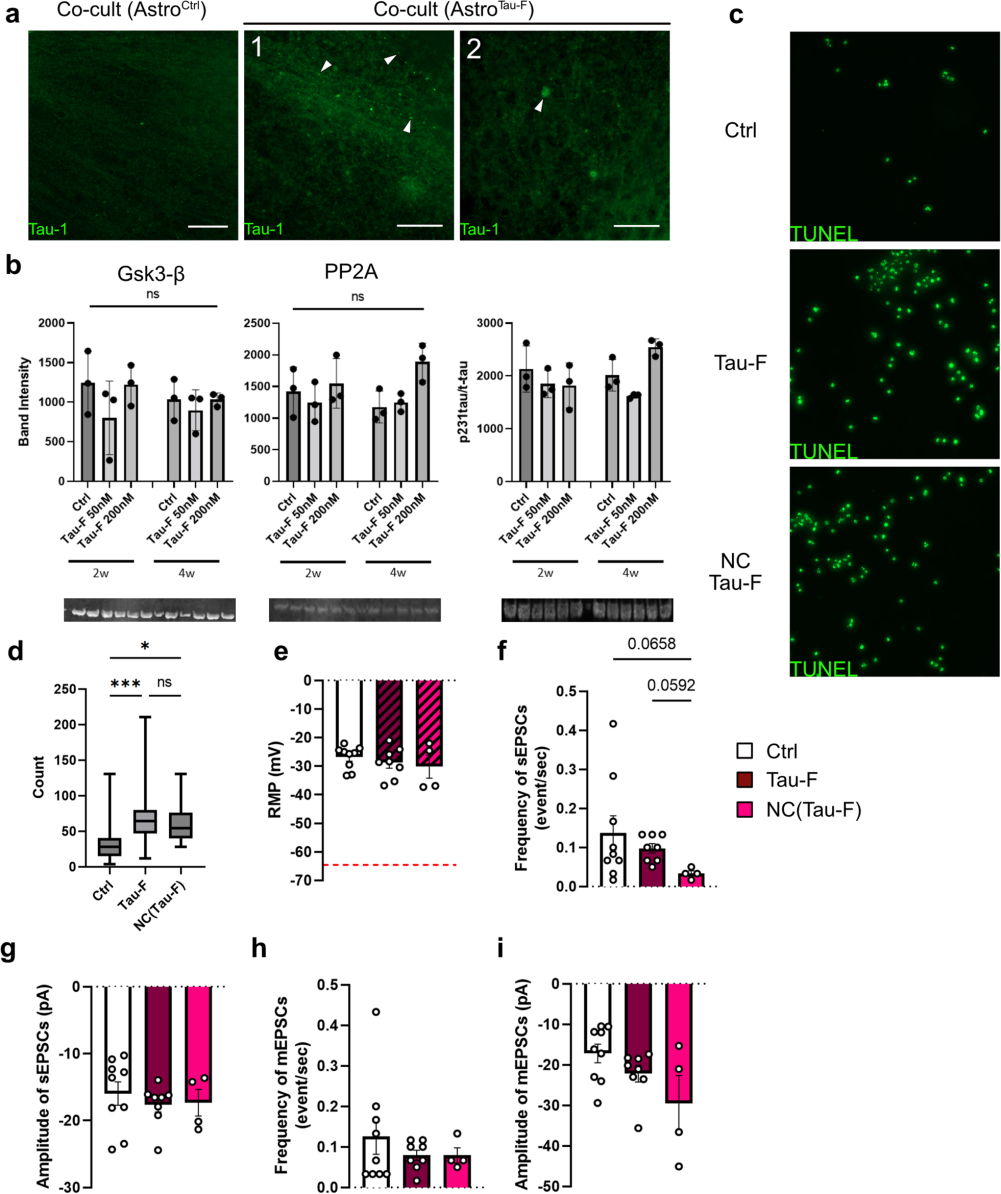
Astrocytes with intracellular Tau-deposits are toxic to neurons in a long-term co-culture set-up. **a** Four weeks of co-culturing with Tau-F-containing astrocytes, resulted in various forms of endogenous tau deposits in neurons (white arrows). The major types of tau deposits found in the neurons that have been co-cultured with tau-exposed astrocytes were strings of small bead shaped aggregates (1) and spherical clusters (2). **b** WB analysis of the four-week co-cultures using antibodies for Gsk3-β, PP2A and pTau231. **c** TUNEL staining of co-cultures where astrocytes have been pre-exposed to either Tau-F or neuronal corpses with tau pathology (NC Tau.F). **d** Quantification of TUNEL positive nuclei/field. **e**–**i** Patch-clamp readings of neurons in co-culture with control astrocytes or astrocytes that had been exposed to Tau-F or neuronal corpses with tau pathology. The graphs show **e** the resting membrane potential relative -65 mV, **f** sEPSC frequency **g** sEPSC amplitude **h** mEPSC frequency **i** mEPSC amplitude. Traces for patch-clamp readings are shown in Additional file 7: Fig. S6. Scale bars = 50 µm. Statistical analysis in b and d was performed using Kruskal Wallis test. Details about the statistical analysis for the electrophysiology data in **e**-**i** is outline in Additional file 9: Table.S2. *p < 0.05, **p < 0.01, ***p < 0.005, ****p < 0.0001

Considering that astrocytes are able to transmit engulfed tau-aggregates, we next investigated if astrocytes with tau deposits could induce tau pathology in healthy neurons. We found that neurons co-cultured with astrocytes pre-exposed to Tau-F, indeed displayed signs of tau distribution (Fig. 6a). Various forms of endogenous tau deposits were present, including strings of small bead shaped aggregates and spherical clusters (Fig. 6 TNT a), closely resembeling those previously observed in monocultures of neurons exposed directly to Tau-F (Fig. 2b). However, WB analysis did not display the same changes in GSK3-β, PP2A or phospho-tau (Fig. 6b) as shown in the neuronal monocultures analysis. Since we were unable to detect any change with AT8 or pSer400 stainings, we used antibodies for pTau231 (an earlier pathology marker). However, even pTau231 did not display any statistically significant change (Fig. 6b). To find out if astrocytic storage of cell corpses with tau pathology alter the overall viability or neuronal activity in a neuronal:astrocyte co-culture, we performed TUNEL assays (Fig. 6c, d) and electrophysiology analysis (Fig. 6e–i). Quantification of TUNEL positive cells showed that viability was clearly affected, when neurons were co-cultured with astrocytes that had been exposed to dead cells. However, we did not detect any statistically significant difference between “control” dead neurons and dead neurons with tau pathology (Fig. 6d), indicating that this effect is not tau dependent, but instead more likely driven by a general overload caused by cellular debris.

The impact of astrocytes on neuronal function was determined by patch-clamp recordings of synaptic currents. As noted before by us and others, hiPS-derived cells display a depolarized resting membrane potential compared to in vivo condition or primary cell cultures. However, there was no difference in membrane potential in corpse-burdened astrocytes compared to controls (Fig. 6e). Our analysis of excitatory postsynaptic currents (sEPSC) revealed a trend for reduction of frequency in debris exposed astrocytes, whereas their amplitude remained unchanged (Fig. 6f–g). Readings of mEPSCs did also not reveal any statistically significant difference between the groups (Fig. 6h–i). In contrast to mEPSC, the sEPSC is a result of endogenous network activity. Considering that the decrease in sEPSC seem to be debris dependent rather than tau dependent, our results indicate that debris-burdened astrocytes have a general cellular effect rather than a direct synaptic one.

### Tau-F storage in astrocytes causes secretion of tau proteoforms with elevated seeding capacity

**Fig. 7:**
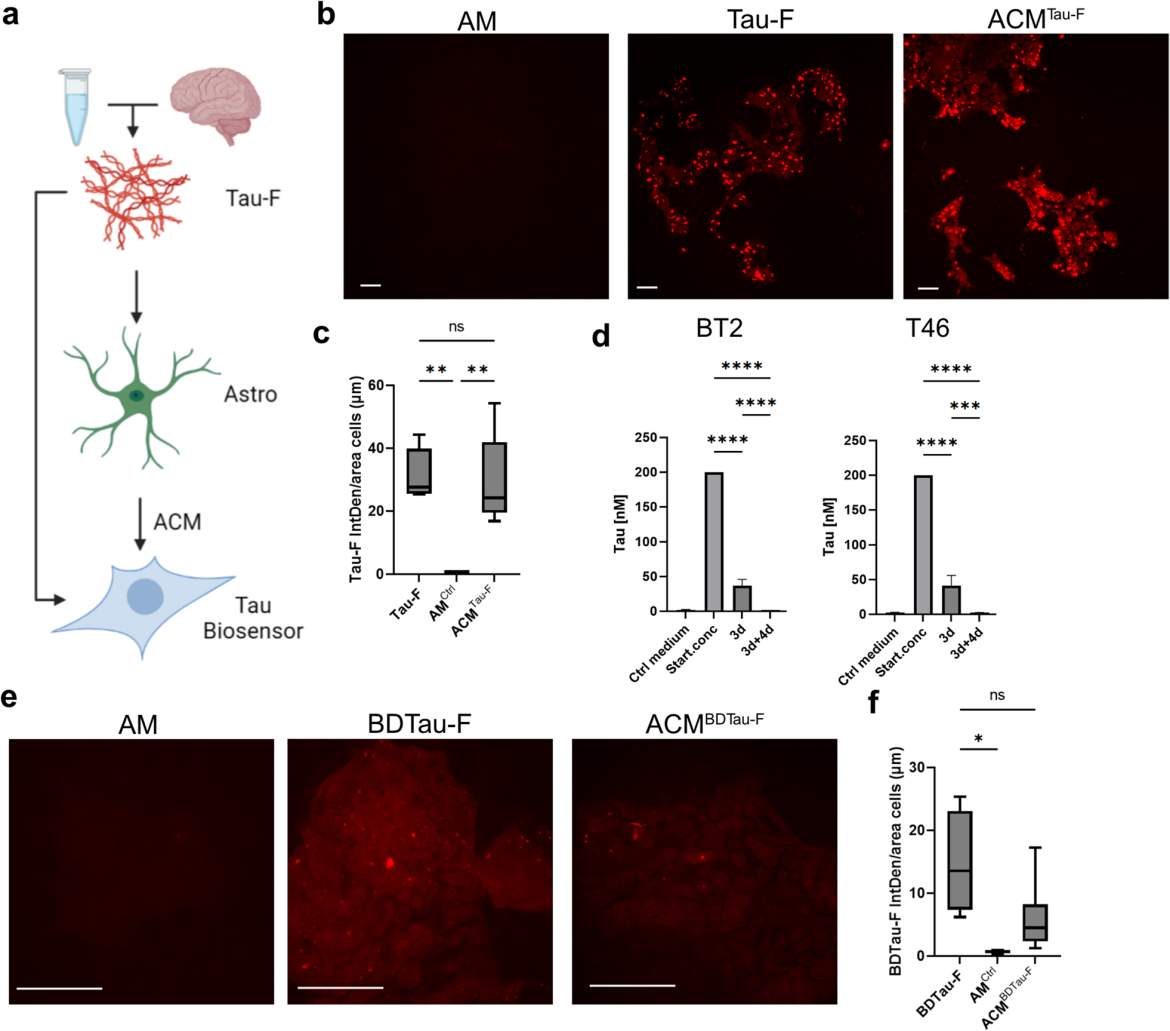
Astrocytes with deposits of synthetic or brain-derived tau secrete tau proteoforms with elevated seeding capacity. Evaluation of tau seeding efficacy using the RD tau P301S FRET biosensor (HEK293T cells). **a** Schematic outline of the experimental set-up. Astrocytes were exposed to 200 nM Tau-F or equivalent levels of human AD brain derived tau (BDTau-F) for 3 days, and incubated for 4 days in tau-free medium (3d + 4d). Tau biosensor cells were then exposed to either Tau-F directly, astrocyte medium from unexposed astrocytes (AM) or astrocyte conditioned medium (ACM). **b** Representative YFP-intensity images of biosensor cells exposed to medium from control astrocytes, Tau-F or ACM^Tau−F^. **c** Quantification of YFP signal from images in b. **d** Quantification of tau in ACM of Tau-F exposed astrocytes by BT2 and T46 ELISA. **e** Representative images from biosensor cells exposed to control astrocyte medium, BDTau-F (direct exposure to brain derived tau diluted in astrocyte medium) or ACM^BDTau−F^ (astrocytes conditioned medium from BDTau-F exposed astrocytes). **f** Quantification of YFP signal from images in e. YFP emission using a 405 nm laser to excite the cells was captured using an LSM700 confocal microscope. YFP IntDen was normalized to the area covered by cells/field of view. Scale bars = 50 µm. One-way ANOVA was used for stastical analysis in c, whilst Kruskal–Wallis test was used in d and f. *p < 0.05, **p < 0.01, ***p < 0.005, ****p < 0.0001

Astrocytes are known to be secretory cells. Hence, we next investigated if astrocytes with deposits secrete any tau species into the medium that can seed tau pathology. A FRET-based tau biosensor cell line was exposed to astrocyte conditioned medium (ACM) from control astrocytes or astrocytes that had been exposed to Tau-F or BDTau-F (Fig. 7a). The medium was collected at 3d + 4d, meaning that the biosensor cells were only exposed to material secreted by the astrocytes during the 4 days incubation in tau-free medium. As expected, virtually no positive YFP-signal was detected in the tau biosensor cell cultures exposed to astrocyte control medium (AM), while direct Tau-F exposure resulted in distinct inclusions of endogenously aggregated protein (Fig. 7b). Strikingly, the YFP signal in biosensor cells exposed to the ACM from Tau-F exposed astrocytes (ACM^Tau−F^) was comparable to that of its positive control (Fig. 7c), Although we know from the intracellular quantifications (Fig. 3) that only a very small fraction of the originally internalized Tau-F is secreted. To assess the extent of tau secretion, we next performed ELISA on conditioned medium from tau exposed astrocytes. Different sandwich ELISA set-ups resulted in tau levels that were indistinguishable from that of control astrocytes that had not been exposed to tau (Fig. 7d). These results confirm that astrocytes although secreting low levels of tau, cause widespread tau seeding, indicating that the secreted tau species have an extreme seeding capacity. Moreover, we observed the same phenomenon when the astrocytes were exposed to BDTau-F (Fig. 7e–f), indicating that astrocytes handle AD brain derived tau species in a similar way as the synthetic tau fibrils. As a whole, this leads us to reason that the individual excreted tau aggregates must possess a greater seeding capacity compared to the original Tau-F/BDTau-F engulfed by the cells.

## Discussion

Astrocytic tau deposits are frequently found in tauopathies, including AD [9]. However, the origin of these inclusions and their significance with respect to pathology are poorly understood. In this study, we investigated if phagocytic astrocytes have the capacity to act as distributors of tau seeds and thereby spread pathology from one cell to another. Our previous data demonstrate that astrocytes effectively take up large amounts of aggregated proteins, as well as entire dead cells [11, 12, 17, 18, 22, 30]. However, compared to professional phagocytes, such as macrophages and microglia, astrocytes are exceptionally poor at degrading the ingested material^.^[11, 14, 18, 22, 31]. This led us to reason that dead cells, engulfed by astrocytes, may constitute a tau reservoir that could be relevant for the propagation of pathogenic aggregates. To test this hypothesis, we designed an experimental set-up based on hiPSC-derived cell cultures, in which astrocytes were exposed to apoptotic neurons, with or without tau pathology, and then added to healthy neurons. In line with our previous data, that murine astrocytes effectively ingest apoptotic cells that are stored for a very long time [11], we demonstrated that human iPSC-derived astrocytes are capable of engulfing neuronal cell corpses, and continuously do so when the dead cells are present in their vicinity. Furthermore, the astrocytes show no signs of degrading the ingested material within the experimental timeframe.

It has previously been shown that exogenously added tau aggregates can seed pathology in both murine and human neurons [32-35]. Hence, in order to induce endogenous tau pathology in the neurons, prior to apoptosis, we tested to expose the neurons to synthetic seeds of Tau-F or Tau-O. Hyperphosphorylated tau is a major pathological hallmark of AD. For instance, pS202/pT205 (recognized by the AT8 antibody) have been used as the quintessential pathology marker for a long time. However, it is still unclear exactly which phospho-variants of tau that are critical for pathology. Several studies have provided evidence that an overall increase in phosphorylation predates tau aggregation [36, 37]. Our WB analysis of cell lysates, using AT8 and pSer400 antibodies, demonstrated that Tau-F exposed neurons contained phosphorylated tau, as well as increased levels of the kinase Gsk3-β. Gsk3-β is a ubiquitous serine/threonine kinase that directly phosphorylates tau and is known to be upregulated in AD patients [38-41]. The combination of increased levels of the Gsk3-β enzyme and general phosphorylated variants of tau protein are strong indicators of developing tau pathology. These changes were not detected in neurons that had been exposed to Tau-O, which could be explained by a higher toxicity, leading to neuronal death before the seeds have time to affect the endogenous tau protein. Based on this data, that the Tau-F possess greater seeding capacity, we decided to use Tau-F for the remaining experiments.

Under physiological conditions, the tau protein is actively transported to the axons following transcription. Axonal tau is then hindered to diffuse back into the soma by the so-called tau diffusion barrier (TDB) [42]. In AD, NFTs are formed in the soma of neurons, possibly as a result of defective tau distribution. In line with this hypothesis, it has been demonstrated that pathogenic protein aggregates can damage the TDB, allowing axonal tau to diffuse into the soma [42]. Considering that only a small fraction of the added Tau-F are internalized by the neurons this effect could be a general stress response rather than a direct seeding event. Some evidence for this is that tau has been shown to aggregate in murine neurons as an acute response to herpes simplex virus 1 infection [43]. In 2017, Zempel et al. proposed a mechanism of tau redistribution in neurons, in which Aβ affects the TDB and thereby causes retrograde mobility of axonal tau protein [44]. The same publication also reported that an overexpression of Gsk3-β leads to an impairment of the barrier. To investigate if Tau-F exposure of hiPSC-derived neurons results in tau redistribution, we performed immunocytochemistry experiments using the total tau antibody Tau-1. Interestingly, the pattern of endogenous tau changed dramatically due to exposure to Tau-F seeds, represented as spherical shapes of endogenous tau. To exclude that the dense spherical tau clusters were a result of dying neurons, we performed immunostainings with synaptophysin antibodies and TUNEL labeling. Our results showed that there was no increase in neuronal cell death or synapse impairment in Tau-F exposed neurons, compared to control neurons, indicating that the deposits were a result of tau redistribution in intact, living neurons.

To elucidate how human astrocytes cope with intracellular tau aggregates, we exposed them directly to Tau-F. In line with our previous studies of Aβ and α-synuclein aggregates, we noted that astrocytes engulf Tau-F as long as it is available in the medium [18]. Interestingly, the astrocytes packed and processed the internalized Tau-F in a way that the tau deposits became almost undetectable to antibodies by ICC. This means that tau pathology in astrocytes may go unnoticed in pathological evaluations of AD brain tissue. In a recent publication, Bengoa-Vergniory et al identified very early intracellular tau deposits in various brain cell types, including astrocytes, using proximity ligation assay (PLA) [45]. The future development of antibodies that effectively detect astrocytic tau-deposits may change the view of the AD pathology. Indeed, analysis of the Cy3 signal in astrocytes exposed to Cy3-labeled Tau-F demonstrated that virtually no degradation of tau aggregates occurred during the 6-day culturing period in tau-free medium. Instead, smaller aggregates were concentrated into larger inclusions that were stored intracellularly. This was further supported by WB analysis, demonstrating large amounts of tau in astrocyte lysates several weeks after the tau-exposure. Moreover, the tau-containg astrocytes displayed less LAMP-1 compared to control cells. All of this contribute to idea that astrocytes do not effectively degrade the engulfed tau aggregates. Reactive astrocytes are closely associated to AD pathology [46]. However, this is a complex mechanism and has been proposed to be both protective and detrimental to surrounding cells [47, 48]. Our data show that in response to Tau-F exposure, astrocytes transform into a distinct inflammatory phenotype, with an increased cell volume, reduced process branching and increased GFAP, LAMP-1 and actin expression. During the first 14 days following Tau-F exposure, the number of astrocytes remained stable, but at day 28 the number of cells reduced dramatically compared to control cultures. Meaning, that although the astrocytes try to cope with the Tau-F, by turning into a reactive state they are unable to clear the aggregates and the toxic Tau-deposits, which eventually causes wide spread astrocytic cell death. Using time-lapse microscopy, we could show that dead astrocytes were engulfed by the remaining astrocytes of the culture, which increased their stress load even further. This, most likely, contributes significantly to the increase of LAMP-1 signal observed at the latest time point.

The accumulation of pathogenic proteins in stressed astrocytes may result in cell-to-cell transfer of the aggregates, which could be of relevance for disease propagation [12]. We therefore tested if tau-containing astrocytes were distributing Cy3Tau-F to neighboring cells. Indeed, using live imaging, we could detect an active and efficient transfer of Tau-F via TNTs and direct membrane contact, as well as via engulfment of dead astrocytes. Moreover, by co-culturing Cy3Tau-F exposed astrocytes (donor cells) and healthy BioTracker488 labeled astrocytes (acceptor cells) we confirmed that cell-to-cell transmission of tau deposits between the two cell populations was very effective. After only three days in co-culture, the acceptor astrocytes displayed an equivalent tau signal as the donor astrocytes and the Cy3 signal then remained unchanged throughout the experiment, indicating that astrocytes rapidly distribute tau aggregates amongst each other. To answer whether astrocytes also promote cell-to-cell spreading of tau aggregates to neurons, we performed co-culture experiments with human iPSC-derived neurons and Tau-F pre-exposed astrocytes. Importantly, we found that neurons co-cultured with Tau-F astrocytes displayed the same pathological tau redistribution as neurons that were directly exposed to Tau-F. We reason that the line of bead-like tau clusters seen along the processes of neurons in the co-culture could be an early state of redistribution, in which endogenous tau forms smaller clusters before being transported to the soma, where they deposit in larger, spherical tau clusters. Both, the ingestion of Tau-F or neuronal corpses with tau pathology, make the astrocytes toxic to neurons, indicating that astrocyte modify normal tau to become pathogenic. Interestingly, electrophysiology recordings revealed a difference in excitatory postsynaptic currents (sEPSC) in astrocytes exposed to neuronal corpses. This effect is likely a general cellular effect rather than a direct synaptic one, since the mEPSCs were unaffected.

To further explore the seeding ability of astrocytic tau inclusions we included human brain derived tau and performed experiments with a RD tau P301S FRET biosensor cell line. It has been suggested that Cy3-labelling may reduce the seeding effect of tau aggregates [49]. Therefore, we choose to use unlabeled tau-fibrils for the seeding experiments. Interestingly, the ACM from tau-containing astrocytes induced a very strong FRET signal with clear inclusions of endogenous protein, comparable to the respective positive control. Since we know that the intracellular tau deposits in astrocytes remain intact after 7d in culture, we can assume that the concentration of Tau-F in the conditioned media is extremely low. To assess this further we performed ELISA on the conditioned medium from tau exposed astrocytes. Different sandwich ELISA set-ups resulted in undetectable tau levels. This could mean either that the levels are incredibly low or that the protein excreted are modified in a way that the assay no longer recognizes the target epitope. However, taken together with the ICC-data demonstrating tau-Cy3 positive deposits inside astrocytes weeks after the actual exposure, as well as the positive WB signal, we conclude that the astrocytes contain a large amount of tau even after long periods in culture and that the medium concentration are significantly lower than the starting concentration. This leads us to reason that the excreted tau aggregates must posses a greater seeding capacity relative the native protein the cells were originally exposed to. Notably, the same result was observed when astrocytes were exposed to tau species from human brain material, indicating that they handle tau aggregates from the AD brain in a similar way as the synthetic fibrils. However, further studies would be required to ascertain the specific modifications introduced by astrocytes that affect tau seeding capacity.

Neurodegenerative diseases, including AD, are defined by a loss of brain homeostasis, which could be explained, at least in part, by the fact that severely stressed astrocytes are unable to fulfill their normal tasks. In addition, accumulating evidence indicates that astrocyte may play an active role in the spreading of tau pathology. Taken together, our data show that astrocytes, because of their ineffective degradation of ingested neuronal corpses and tau aggregates, promote cell-to-cell spreading of pathological tau and directly negativly affect the surrounding neurons.

## Supplementary Information

**Additional file 1. **Movie 1Cy3Tau-F inclusions were observed traveling through the TNTs between cells.

**Additional file 2. Fig. S1. **Human iPSC derived neurons and astrocytes express cell type-specific markers.

**Additional file 3. Fig. S2. **Human iPSC derived neurons take up sonicated Tau-F.

**Additional file 4. Fig. S3. **Sonicated tau fibrils display no toxic effects on human iPSC-derived neurons.

**Additional file 5. Fig. S4. **Western blots of astrocytes exposed to Tau-F for 3+11 days.

**Additional file 6. Fig. S5. **Tau-F deposits transfers between astrocytes both through clearance of dying cells and tunneling nanotubes.

**Additional file 7. Fig. S6. **Traces from patch-clamp experiments.

**Additional file 8. Table. S1. **List of all antibodies used in the study.

**Additional file 9.  Table. S2. **Statistical tests for patch-clamp experiments.

**Additional file 10. **Full western blots and loading controls.

## Data Availability

Not applicable.
