# Supplementary material for: Astrocytic uptake of neuronal corpses promotes cell-to-cell spreading of tau pathology
Source: Acta Neuropathol Commun. 2023 Jun 17;11:97. doi: 10.1186/s40478-023-01589-8 (PMC10276914; doi:10.1186/s40478-023-01589-8)
Supplement: Supplementary file 2 — Additional file 2. Fig. S1. Human iPSC derived neurons and astrocytes express cell type-specific markers. [file 40478_2023_1589_MOESM2_ESM.pdf]

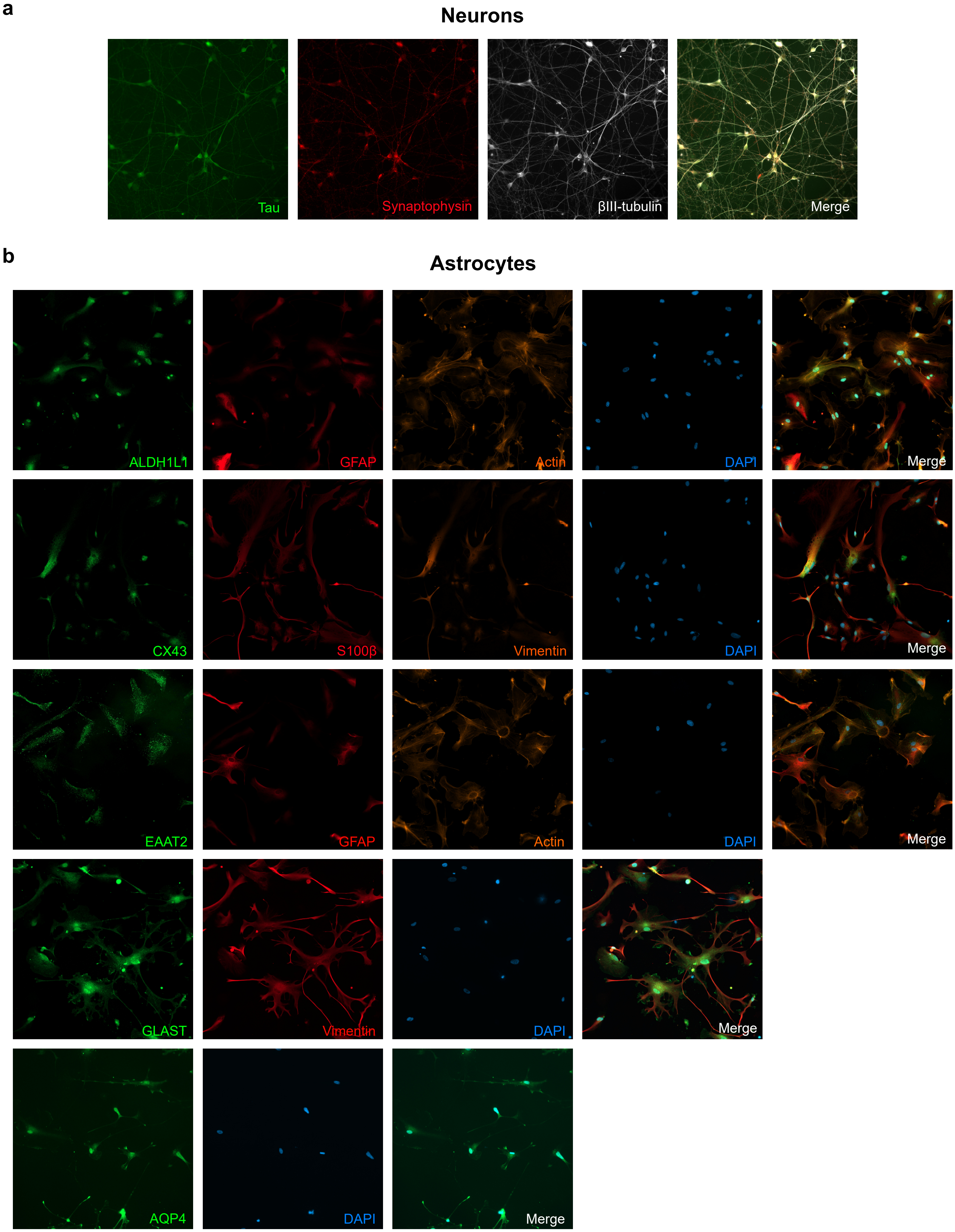

Fig.S1 Human iPSC derived neurons and astrocytes express cell type-specific markers. (a) Expression of neuron specific markers. Human iPSC derived neurons express several neuronal markers including tau, synaptophysin and βIII-tubulin. (b) Expression of astrocyte specific markers. Human iPSC derived astrocytes express a plethora of markers including ALDH1L1, CX43, EAAT2, GLAST, AQP4, GFAP, S100β and vimentin. Phalloidin and DAPI stain the actin and nucleus respectively.
