# Supplementary material for: Astrocytic uptake of neuronal corpses promotes cell-to-cell spreading of tau pathology
Source: Acta Neuropathol Commun. 2023 Jun 17;11:97. doi: 10.1186/s40478-023-01589-8 (PMC10276914; doi:10.1186/s40478-023-01589-8)
Supplement: Supplementary file 3 — Additional file 3. Fig. S2. Human iPSC derived neurons take up sonicated Tau-F. [file 40478_2023_1589_MOESM3_ESM.pdf]

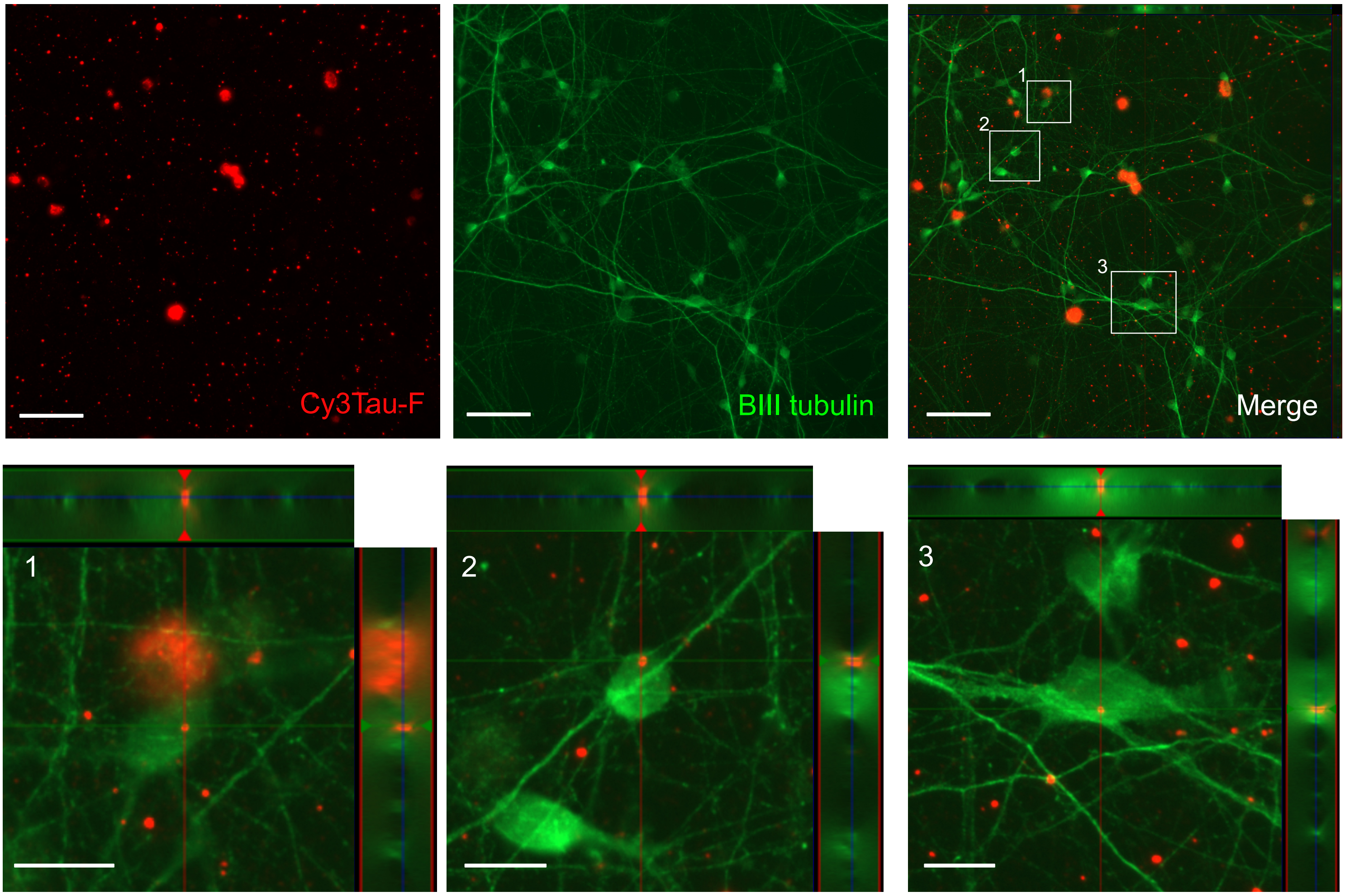

Fig.S2 Human iPSC derived neurons take up sonicated Tau-F. Representative images of Cy3Tau-F exposed neuronal mono-cultures illustrate neuronal internalization of Tau-F aggregates. Scale bars for whole image is set to 50 µm and 10 µm for area 1, 2 and 3.
