# Supplementary material for: Astrocytic uptake of neuronal corpses promotes cell-to-cell spreading of tau pathology
Source: Acta Neuropathol Commun. 2023 Jun 17;11:97. doi: 10.1186/s40478-023-01589-8 (PMC10276914; doi:10.1186/s40478-023-01589-8)
Supplement: Supplementary file 4 — Additional file 4. Fig. S3. Sonicated tau fibrils display no toxic effects on human iPSC-derived neurons. [file 40478_2023_1589_MOESM4_ESM.pdf]

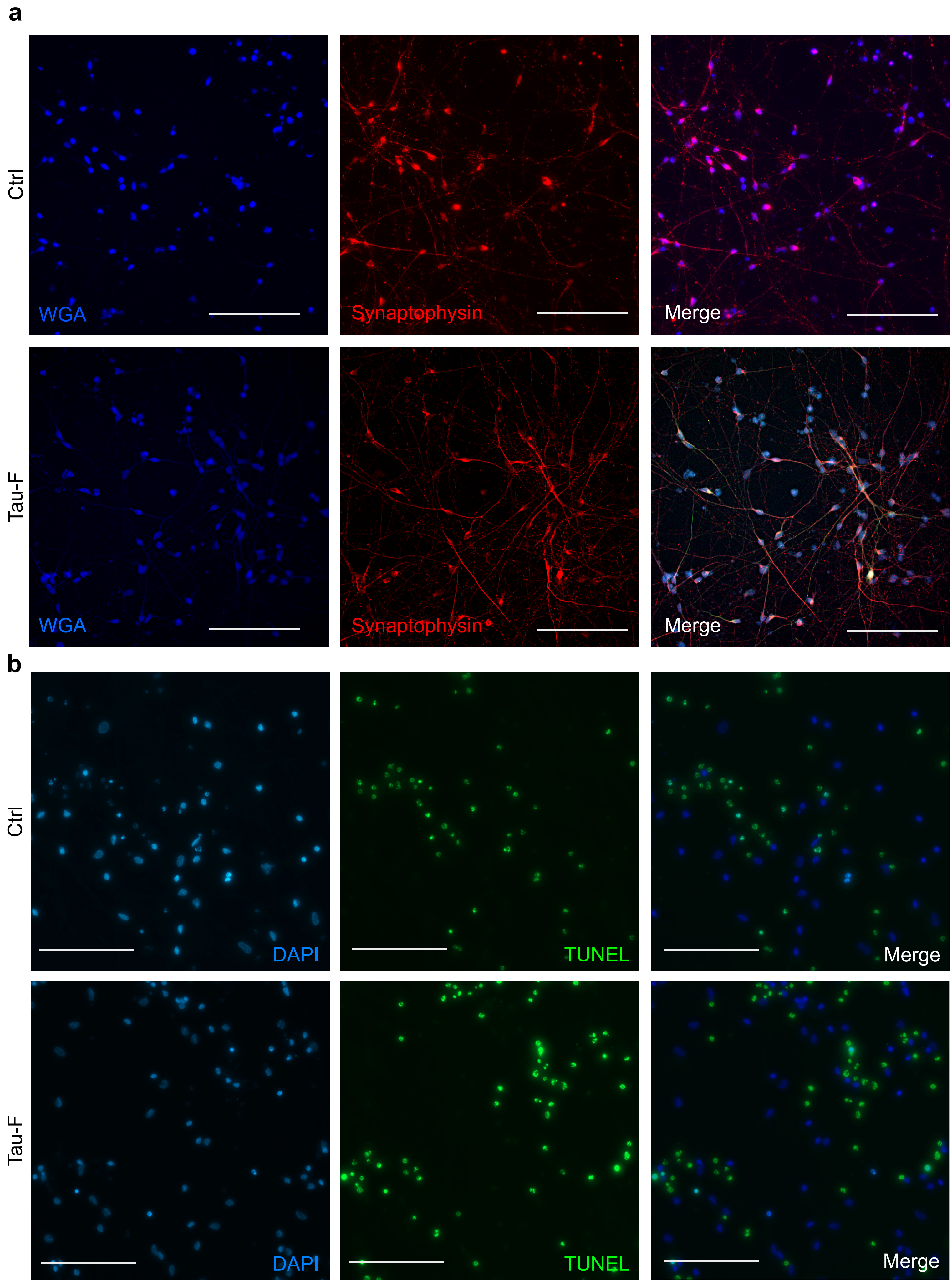

Fig.S3 Sonicated tau fibrils display no toxic effects on human iPSC-derived neurons. Ctrl and Tau-F exposed neuronal mono-cultures 4 weeks post-exposure. (a) Neurons stained with WGA and Synaptophysin. (b) Neurons stained with DAPI and TUNEL. Scale bars set to 100 µm.
