# Supplementary material for: Astrocytic uptake of neuronal corpses promotes cell-to-cell spreading of tau pathology
Source: Acta Neuropathol Commun. 2023 Jun 17;11:97. doi: 10.1186/s40478-023-01589-8 (PMC10276914; doi:10.1186/s40478-023-01589-8)
Supplement: Supplementary file 5 — Additional file 5. Fig. S4. Western blots of astrocytes exposed to Tau-F for 3+11 days. [file 40478_2023_1589_MOESM5_ESM.pdf]

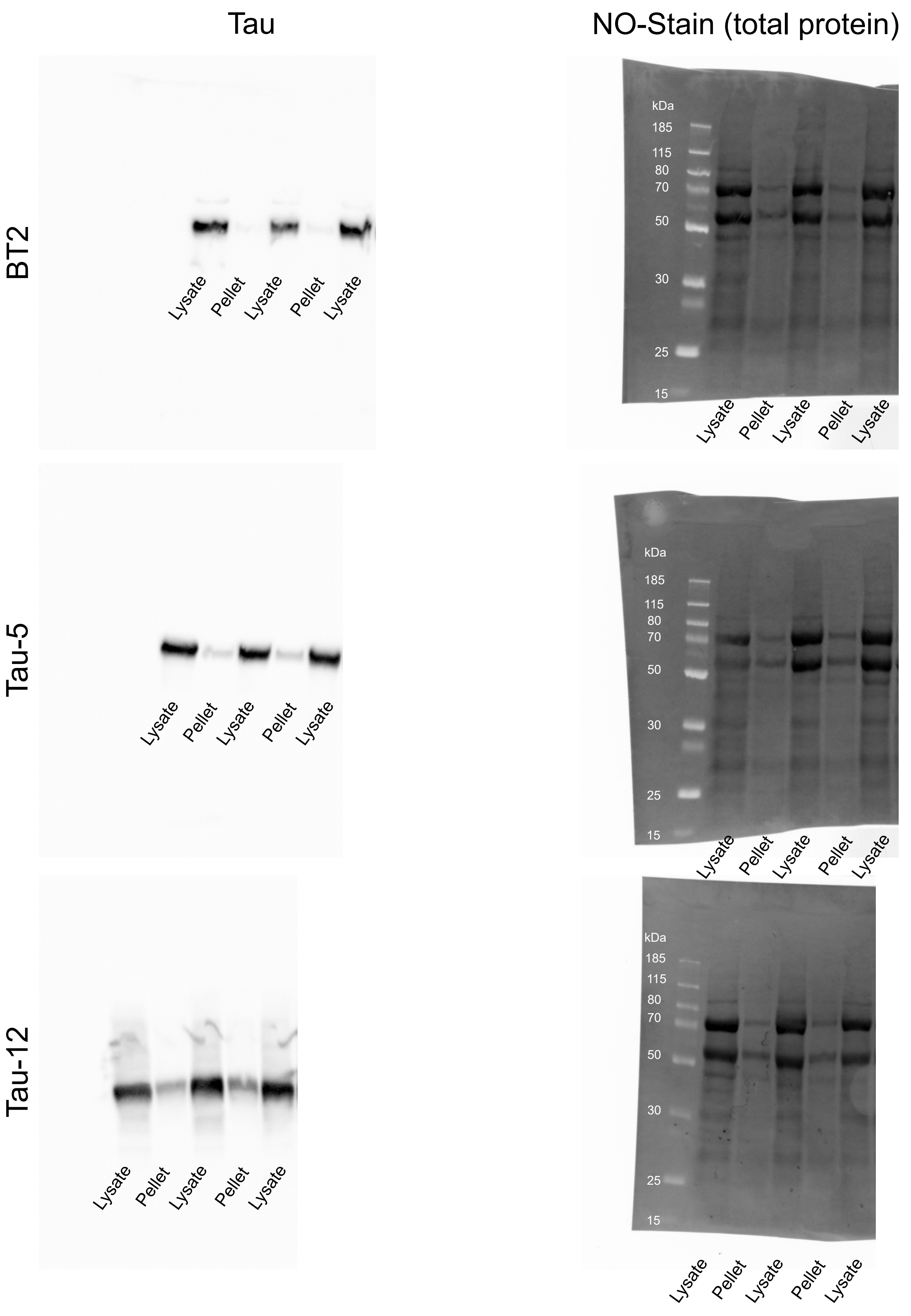

Fig.S4 Western blots of astrocytes exposed to Tau-F for 3+11 days. Antibodies used were: BT2 (aa 194-198), Tau-5 (aa 218-225) and Tau-12 (aa 6-18). The blots to the right are the corresponding total protein for the same membrane.
