# Supplementary material for: Astrocytic uptake of neuronal corpses promotes cell-to-cell spreading of tau pathology
Source: Acta Neuropathol Commun. 2023 Jun 17;11:97. doi: 10.1186/s40478-023-01589-8 (PMC10276914; doi:10.1186/s40478-023-01589-8)
Supplement: Supplementary file 6 — Additional file 6. Fig. S5. Tau-F deposits transfers between astrocytes both through clearance of dying cells and tunneling nanotubes. [file 40478_2023_1589_MOESM6_ESM.pdf]

a

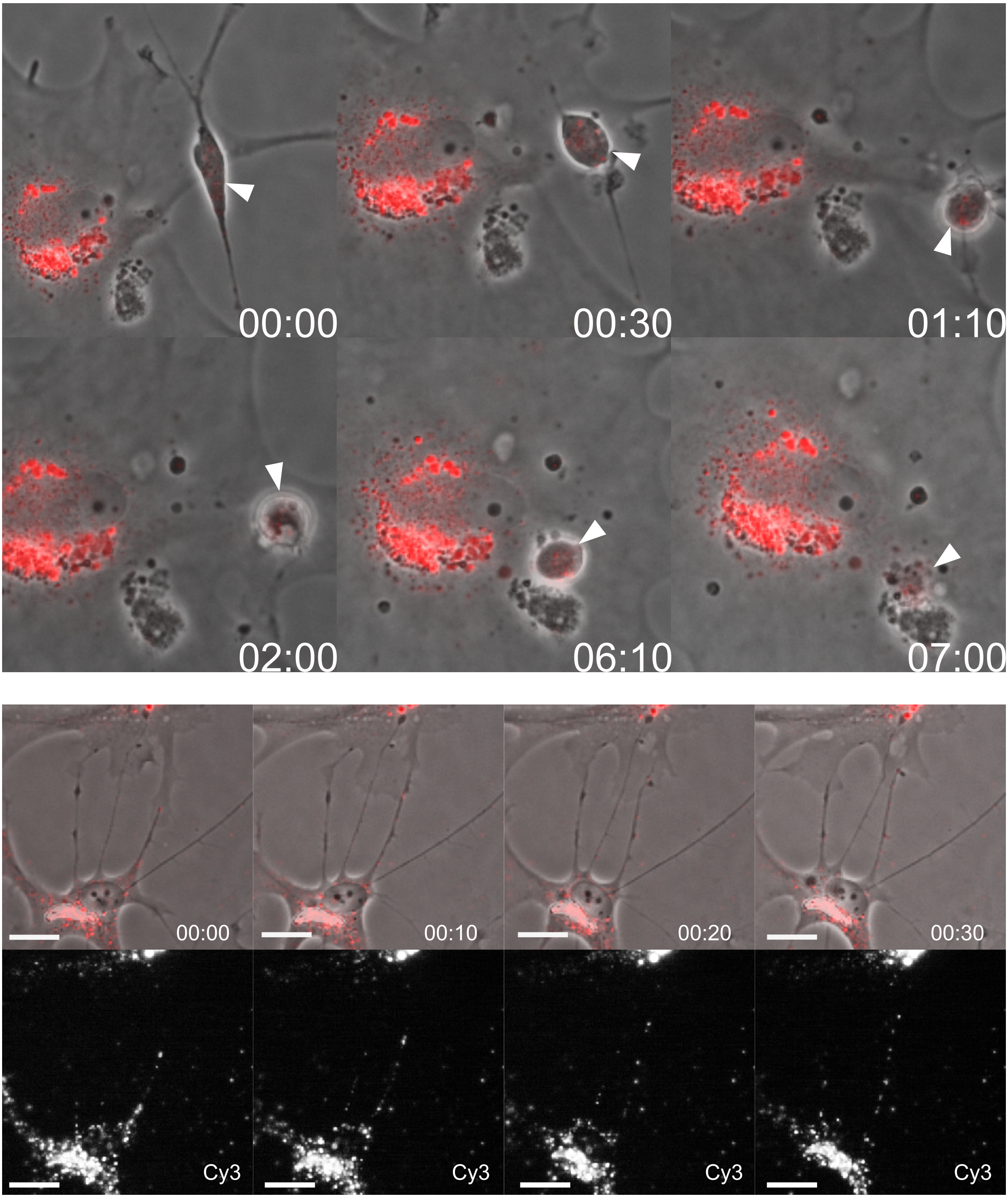

Fig.S5 Tau-F deposits transfers between astrocytes both through clearance of dying cells and tunneling nanotubes. (a) A dying Cy3Tau-F positive astrocyte (indicated by the white arrows) being engulfed and subsequently processed by a neighboring astrocytes over the period of 7 hours. (b) TNT-mediated transfer of tau aggregates between astrocytes. Scar bars=20μm.
