# Supplementary figures and images for: Astrocytic uptake of neuronal corpses promotes cell-to-cell spreading of tau pathology
Source: Acta Neuropathol Commun. 2023 Jun 17;11:97. doi: 10.1186/s40478-023-01589-8 (PMC10276914; doi:10.1186/s40478-023-01589-8)

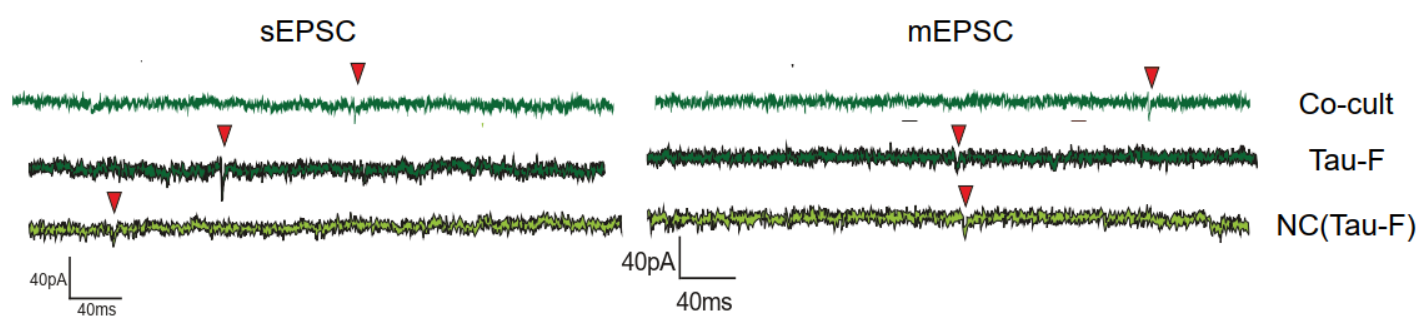

**Fig.S6** Traces from patch-clamp experiments.

Supplement: Supplementary file 7 — Additional file 7. Fig. S6. Traces from patch-clamp experiments. [file 40478_2023_1589_MOESM7_ESM.pdf]

Fig.2 a Antibody

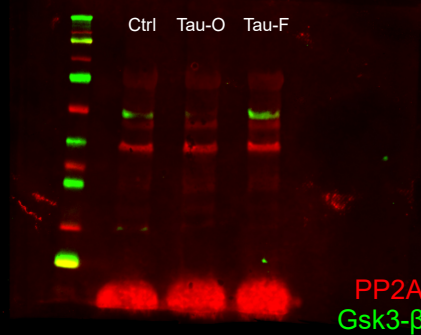

No-Stain

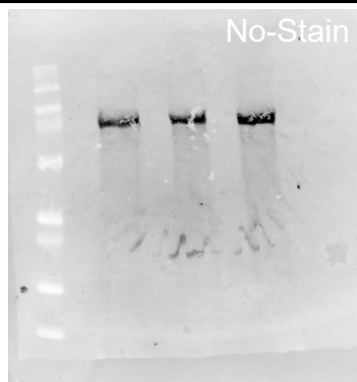

Fig.2 a

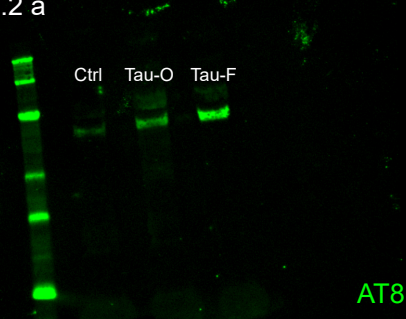

Fig.2 a

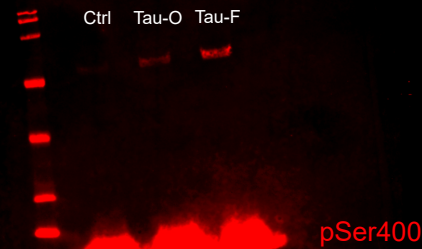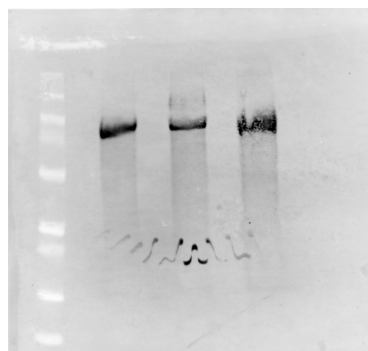

Fig.6 b

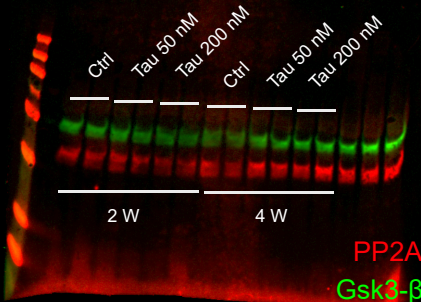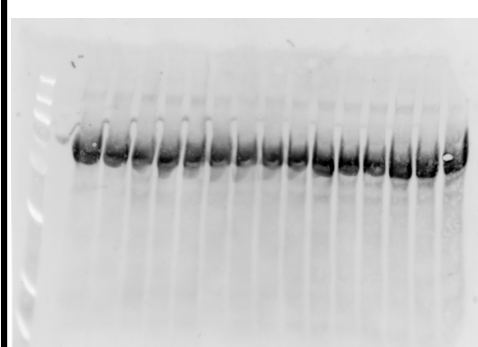

Fig.6 b

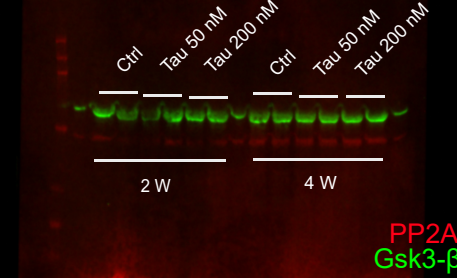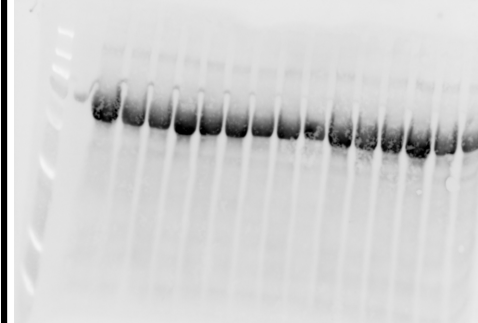

Fig.6 b

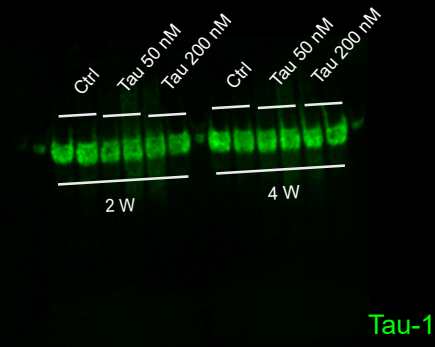

Antibody

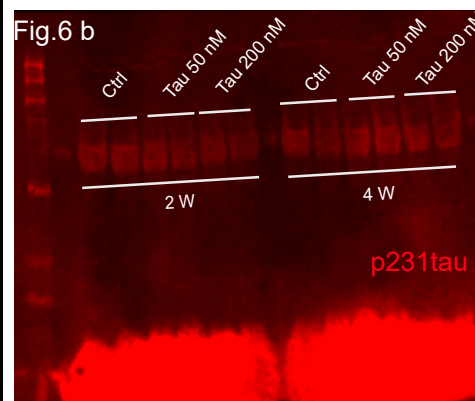

NO-Stain

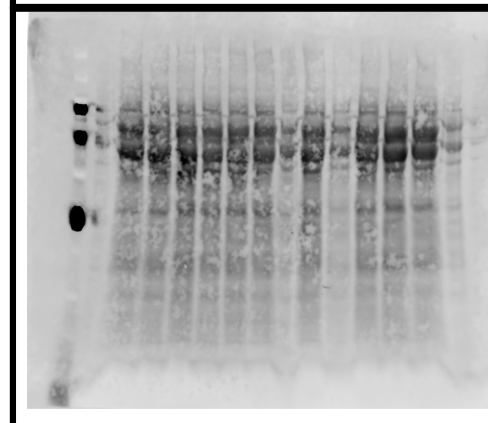

Supplement: Supplementary file 10 — Additional file 10. Full western blots and loading controls. [file 40478_2023_1589_MOESM10_ESM.pdf]
