# Supplementary material for: Astrocytic uptake of neuronal corpses promotes cell-to-cell spreading of tau pathology
Source: Acta Neuropathol Commun. 2023 Jun 17;11:97. doi: 10.1186/s40478-023-01589-8 (PMC10276914; doi:10.1186/s40478-023-01589-8)
Supplement: Supplementary file 8 — Additional file 8. Table. S1. List of all antibodies used in the study. [file 40478_2023_1589_MOESM8_ESM.pdf]

Manufacturer	Antibody/Dye	Target	Dilution
BD Biosciences (610201)	7/GSK-3b	Gsk3-β	WB 1:2500
ThermoFisher (PA5-17510)	PP2A	PP2Aα	WB 1:2500
ThermoFisher (MN1020	AT8	Tau (pSerine202/pThreonine 205)	WB 1:2500<br>ICC 1:400
Abcam (ab151559)	EPR2488	Tau (pSerine231)	WB 1:2500
Anaspec (AS-54978	pSer400	Tau (pSerine400)	WB 1:2500
SigmaAldrich (Mab3420)	Tau-1 (PC1C6)	Tau (total)	WB 1:2500<br>ICC 1:200
SigmaAldrich (SCT106)	BioTracker488	Cellular membranes	1:200
Biolegend (BT-RTQOJH-50)	Tuj1	βIII-tubulin	ICC 1:500
Abcam (ab3427)	EP1097i	Synaptophysin	ICC 1:400
Abcam (ab20346)	VI-10	Vimentin	ICC 1:400
Abcam (ab87117)		ALDHL1L1	ICC 1:200
AH diagnostics (SC-365634)	E-1	EAAT2	ICC 1:200
Santa Cruz (SC-32739)		AQP4	ICC 1:100
Sigma (S2532)		S100β	ICC 1:200
Invitrogen (35568)	DyLight 700	Goat anti-Rb	WB 1:20 000

ThermoFisher (Sa5-35571)	DyLight 800	Goat anti-Rb	WB 1:20 000
Invitrogen (35518)	DyLight 700	Goat anti-Ms	WB 1:20 000
Invitrogen (Sa5-535521)	DyLight 800	Goat anti-Ms	WB 1:20 000
ThermoFisher (A-11001, A-21422, A-21235)	AlexaFluor 488, 555, 647	Goat anti-Ms	ICC 1:200
ThermoFisher (A-11008, A-21244)	AlexaFluor 488, 647	Goat anti-Rb	ICC 1:200
ThermoFisher (A-21449)	AlexaFluor 647	Goat anti-Ch	ICC 1:200
ThermoFisher (MN1010)	BT2	Tau Central (aa 194-198)	WB (1:400)<br>ELISA 1 µg/mL
ThermoFisher (136400)	T46	Tau C-terminus (aa 404-441)	WB (1:400)<br>ELISA 1 µg/mL
BioSite (806501)	Tau-12	Tau N-terminus (aa 6-18)	WB (1:400)<br>ELISA 1 µg/mL

Table.S1 List of all antibodies used in the study
