# Supplementary material for: Astrocytic uptake of neuronal corpses promotes cell-to-cell spreading of tau pathology
Source: Acta Neuropathol Commun. 2023 Jun 17;11:97. doi: 10.1186/s40478-023-01589-8 (PMC10276914; doi:10.1186/s40478-023-01589-8)
Supplement: Supplementary file 9 — Additional file 9. Table. S2. Statistical tests for patch-clamp experiments. [file 40478_2023_1589_MOESM9_ESM.pdf]

Test	Variable	Value	p-value
Student t test comapred to -65mV	Resting membrane potential	Non treatment: 28,070 Tau: 19,132 Tau + debris: 8,657	Non treatment: p<0,001 Tau: p<0,001 Tau + debris: p=0,002
Kruskal-Wallis	Resting membrane potential	0,843	0,656
Kruskal-Wallis	Frequency of sEPSC	6,366	0,041
Kruskal-Wallis	Amplitude of sEPSC	1,295	0,523
Kruskal-Wallis	Frequency of mEPSC	0,162	0,922
Kruskal-Wallis	Amplitude of mEPSC	3,809	0,149
**COEFFICIENT OF VARIATION STATISTICS**			
Asymptotic test	Resting membrane potential	1,5926	0,45
MSLRT		47,3836	<0,001
Asymptotic test	Frequency of sEPSC	4,4969	0,105
MSLRT		4,506733	0,105
Asymptotic test	Amplitude of mEPSC	1,355	0,507
MSLRT		32,6327	<0,001
Pairwise comparisons			
**Value**	**p-value**		
No treatment x debris: -0,312 No treatement x Tau + debris: 8,5 Tau x Tau + Debris: 8,813	No treatment x debris: p=1 No treatement x Tau + debris: p=0,066 Tau x Tau + Debris: p=0,059		

Table.S2 Statistical tests for patch-clamp experiments
